# Extending Bayesian back-calculation to estimate age and time specific HIV incidence

**DOI:** 10.1007/s10985-019-09465-1

**Published:** 2019-02-27

**Authors:** Francesco Brizzi, Paul J. Birrell, Martyn T. Plummer, Peter Kirwan, Alison E. Brown, Valerie C. Delpech, O. Noel Gill, Daniela De Angelis

**Affiliations:** 1 Medical Research Council Biostatistics Unit, School of Clinical Medicine, University of Cambridge, Cambridge, CB2 0SR UK; 2 IARC, 150 Cours Albert Thomas, 69372 Lyon Cedex 08, France; 3 Public Health England, Colindale, London, NW9 5EQ UK

**Keywords:** Back-calculation, Multi-state model, Bayesian inference, Splines, Routinely collected data

## Abstract

CD4-based multi-state back-calculation methods are key for monitoring the HIV epidemic, providing estimates of HIV incidence and diagnosis rates by disentangling their inter-related contribution to the observed surveillance data. This paper, extends existing approaches to age-specific settings, permitting the joint estimation of age- and time-specific incidence and diagnosis rates and the derivation of other epidemiological quantities of interest. This allows the identification of specific age-groups at higher risk of infection, which is crucial in directing public health interventions. We investigate, through simulation studies, the suitability of various bivariate splines for the non-parametric modelling of the latent age- and time-specific incidence and illustrate our method on routinely collected data from the HIV epidemic among gay and bisexual men in England and Wales.

**Electronic supplementary material:**

The online version of this article (10.1007/s10985-019-09465-1) contains supplementary material, which is available to authorized users.

## Introduction

Quantification of HIV incidence and prevalence is key to HIV surveillance and the design and evaluation of targeted interventions. Direct measurement of these quantities is, however, infeasible: infection times are unobserved and, due to the long asymptomatic incubation period, a large proportion of infections remain undiagnosed. Therefore, a number of statistical approaches have been developed to estimate HIV burden from routinely collected surveillance data. The back-calculation method, initially proposed by Brookmeyer and Gail (1987, 1988) still plays a key role in the monitoring of HIV and other long incubation diseases (Deuffic-Burban et al. 2007; Sweeting et al. 2007; van Sighem et al. 2015). The idea underlying this approach is that the infection process can be reconstructed from time series data on disease endpoint events and knowledge of the distribution of the time between infection and the endpoints of interest. For HIV, in a discrete time formulation, this is formally expressed by the convolution equation (Becker et al. 1991):

1 $$\begin{aligned} a_i = \sum _{i_0=1}^{i} h_{i_0} f_{i-i_0}, \qquad i = 1, \ldots , T \end{aligned}$$

where $a_i$ is the expected number of new AIDS diagnoses in the ${i}$th interval, $(t_{i-1},t_i]$, $h_{i_0}$ is the expected number of new infections in $(t_{i_0-1}, t_{i_0}]$, and $f_{i-i_0}$ is the probability of an AIDS diagnosis in the ${(i-i_0)}$th interval after infection.

The back-calculation model (1) has been extended to: incorporate new information and data types to refine incidence estimates (e.g. Aalen et al. 1994; Bellocco and Marschner 2000; Chau et al. 2003; Sweeting et al. 2005; Ndawinz et al. 2011; Yan et al. 2011); and to usefully characterise the incubation period as progression through disease stages of increased severity (e.g. Longini et al. 1992; Dietz et al. 1994; Aalen et al. 1997; Sweeting et al. 2005; Sommen et al. 2009; Birrell et al. 2012).

A further extension, again aimed at providing a better insight into the epidemic, has been to estimate time- and age-specific infection rates, making use of the information that age at infection is the strongest predictor of HIV progression. In principle, this would entail the specification of a latent time- and age-(denoted $i_0$ and $j_0$) specific bivariate surface $h_{i_0,j_0}$, which is particularly challenging to estimate. To avoid modelling a two-dimensional infection surface, Verdecchia and Mariotto (1995), Greenland (1996) and Wand et al. (2009) applied age-independent back-calculation models to diagnosis data stratified by birth-cohorts, deriving age-dependent incidence estimates through the combination of the estimates resulting from each cohort. Also to simplify the problem, some authors (Becker and Marschner 1993; Becker et al. 2003) used a multiplicative model, $h_{{i_0},{j_0}}=h_{i_0} h_{j_0}$ which however cannot capture different time-trends across age-groups. The non-parametric bivariate step-function in Rosenberg (1995) added flexibility to the infection surface model and Marschner and Bosch (1998) improved parameter identifiability by imposing (thin plate spline) smoothing at the corner-points. The level of smoothing in Marschner and Bosch (1998) was however isotropic (i.e. equal in both the time and age dimensions), which may not always be appropriate, for instance when time and age are measured on different scales. All these approaches solely considered counts of AIDS diagnoses as endpoint data, with the exception of Becker et al. (2003) who additionally incorporated HIV diagnoses.

In this paper we reconsider the problem of age specific estimation but in the context of a CD4 count based multi-state model, extending the work of Aalen et al. (1997), Sweeting et al. (2005) and Birrell et al. (2012) to estimate age–time dependent HIV incidence, CD4 state specific diagnosis probabilities and number of undiagnosed infections. We also extend the work of Marschner and Bosch (1998) by adopting bivariate splines (Wood 2006a) to model the incidence surface as a continuous function of age and time and we further investigate tensor product splines (Eilers and Marx 2003; Wood 2006b), allowing for differential smoothing in the time and age dimensions. In contrast to earlier age-dependent back-calculation approaches (e.g. Marschner and Bosch 1998), the use of a multiplicity of data introduces the complication that the back-calculation model cannot be expressed as a generalised linear model (GLM), so that the inferential problem becomes non-standard. We propose a Bayesian approach to estimation, which can more easily tackle the non-standard nature of the problem and automatically allows propagation of uncertainty to all the derived quantities of interest.

Section 2 describes the motivating application and describes the data available from England and Wales, with relevant notation. Section 3 introduces the CD4-staged back-calculation model and links this model to the data. Section 4 looks at a range of spline models suitable for smoothing the incidence surface and the merits of each spline are examined in the simulation study of Sect. 5. In Sect. 6, appropriate model parameterisations are then used to estimate age-stratified HIV incidence in England and Wales over the last 20 years. We conclude with a discussion in Sect. 7.

## Motivating application

**Fig. 1:**
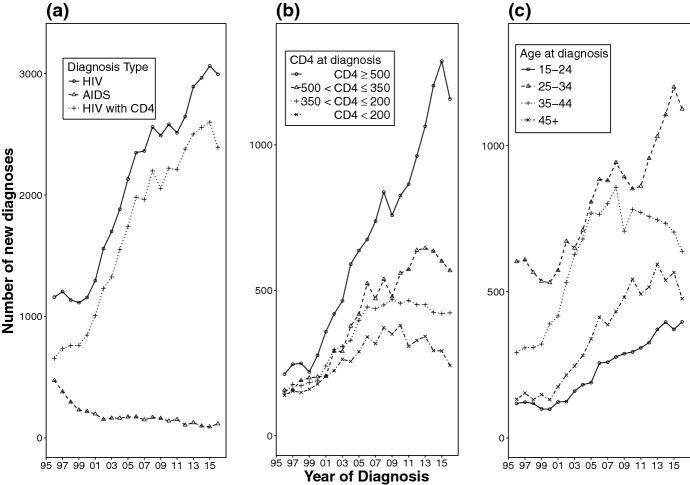
Number of new diagnoses, by year of diagnosis: **a** new diagnoses, by diagnosis type; **b** HIV diagnoses stratified by CD4 count at diagnosis; **c** HIV diagnoses stratified by age at diagnosis

The methodology developed in this paper is motivated by the surveillance data routinely collected by Public Health England (PHE) to monitor the HIV epidemic among men-who-have-sex-with-men (MSM) in England and Wales (see Fig. 1). Available data include diagnoses of HIV over time classified in two groups, according to the presence or absence of AIDS related symptoms within 3 months of the initial HIV diagnosis. These will be loosely expressed as diagnoses of AIDS and HIV respectively. Information on the CD4 cell counts around diagnosis (i.e. taken within 3 months of HIV diagnosis) is also available for a large, and increasing, proportion of the new HIV diagnoses (see Fig. 1a, b).

**Fig. 2:**
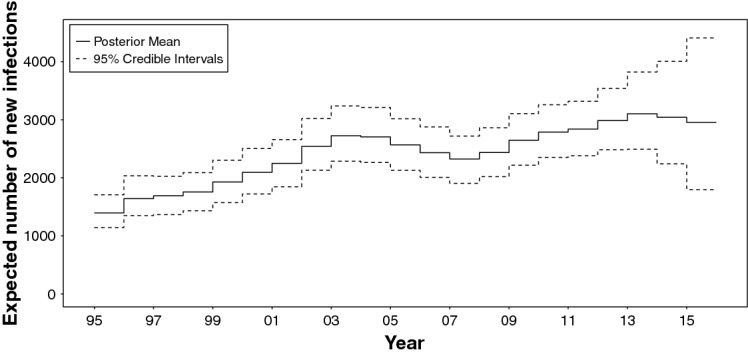
Expected number of new infections estimated using the method of Birrell et al. (2013)

A Bayesian back-calculation analysis of this type of data (Birrell et al. 2012) collected over the whole epidemic history (i.e. 1978–2015), resulted in the estimated yearly number of new HIV infections levelling off at approximately 3000 (see Fig. 2), following a steady increase over the period 2007–2013. However, stratification of new diagnoses by age (Fig. 1c) reveals heterogeneous trends, questioning whether the apparent plateau in incidence might mask contrasting trends in different age-groups, suggesting the need for age specific incidence estimates.

Formally, assume the epidemic period $$(t_0,t_T]$$ is split into T disjoint, consecutive intervals $$(t_{i-1},t_i]$$, $$i=1, \dots , T$$. Similarly the age-range $$(a_0,a_{A}]$$ is subdivided into A disjoint, consecutive groups $$(a_{j-1},a_j]$$, $$j=1, \dots , A$$. We will, in places, refer to $$(t_{i-1},t_i]$$ as the $${i}$$th time interval and $$(a_{j-1},a_j]$$ as the $${j}$$th age group. Let $$y^H_{i,j}$$ and $$y^A_{i,j}$$ denote the observed number of new HIV and AIDS diagnoses in the $${i}$$th time interval and $${j}$$th age group, where the $${A}$$th age-group is formed of all diagnoses at ages greater than $$a_{A-1}$$. The $$K \times 1$$ vector $${\varvec{y}}^{H_C}_{i,j}$$ = ($$\hbox {y}^{H_C}_{i,j,1}$$, $$\hbox {y}^{H_C}_{i,j,2}$$, $$\dots $$, $$\hbox {y}^{H_C}_{i,j,K}$$)$$^T$$ gives the distribution of a subset $$n_{i,j}\;(\le y^{H}_{ij})$$ of the HIV diagnoses with a linked CD4 count, classified into K categories: $$[c_1,\infty )$$, $$[c_2,c_1)$$, $$\dots $$, $$[0,c_{K-1})$$, where $$c_1> c_2> \dots > c_{K-1}$$ are appropriate CD4 thresholds. We further define $${\mathbf {y}}^H = (y^H_{11}, \dots , y^H_{1A}, \dots , y^H_{T1}, \dots y^H_{TA})^T$$ and $${\mathbf {y}}^A = (y^A_{11}, \dots , y^A_{1A}, \dots , y^A_{T1}, \dots y^A_{TA})^T$$ to be $$TA \times 1$$ vectors of the number of new HIV and AIDS diagnoses over time and age respectively, and $${\mathbf {y}}^{H_C} = \{{\varvec{y}}^{H_C}_{1,1}, \dots , {\varvec{y}}^{H_C}_{1,A}, \dots , {\varvec{y}}^{H_C}_{T,1}, \dots , {\varvec{y}}^{H_C}_{T,A}\}$$ to denote the array of CD4-linked diagnoses over time and age.

## Age-dependent multi-state back-calculation

### Model specification

**Fig. 3:**
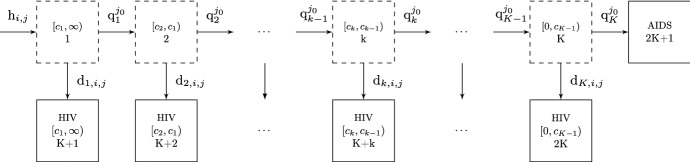
Age-dependent back-calculation multi-state model. Dashed and solid boxes denote undiagnosed and diagnosed states, respectively

The data previously described arise as a result of three distinct, interlinked processes: infection, disease progression and diagnosis. Figure 3 shows the structure of a discrete-time non-homogeneous population-level CD4 count multi-state model that explicitly specifies the contribution of these three processes to the dynamics of an infected population.

The infection process is modelled by a two dimensional non-homogeneous Poisson process (e.g. Rosenberg 1995), with time (*u*) and age (*v*) dependent infection rate $\lambda (u,v)$. Then the expected number of new infections in $(t_{i-1},t_{i}]$ and $(a_{j-1},a_{j}]$ is $h_{i,j} = \int _{t_{i-1}}^{t_{i}} \int _{a_{j-1}}^{a_{j}} \lambda (u,v) \; du dv$. After infection, individuals are subject to competing disease progression and diagnosis pressures, represented by movements to undiagnosed states ($1, \dots , K$) with lower CD4 counts and to the absorbing diagnosis states ($K+1, \dots , 2K+1$), respectively.

Given the discrete time framework, the progression and diagnosis processes are expressed in terms of probabilities. Diagnosis probabilities are state, time- and age-specific, to reflect the weakening of the immune system and to allow for the impact of testing campaigns over time, possibly targeted at specific age groups. Denote $d_{k,i,j}$ the probability of being diagnosed from the *k*th undiagnosed state in the *i*th time and *j*th age group. For those infected in the ${j_0}$th age group and remaining undiagnosed, let $q_{k}^{j_0}$ denote the probability of progressing from the *k*th to the ${(k+1)}$th state in the same interval.

### Model dynamics

Previous work (Aalen et al. 1997; Sweeting et al. 2005; Birrell et al. 2012) has characterised the number of infected individuals in the disease states through a Markov chain. However, in the model of Fig. 3, progression depends on the age at infection. The number of individuals in a state, at a given time and age group no longer depends solely on the number of individuals at the previous time and age, unless the infected population is also stratified by age at infection. This substantially complicates the model dynamics, now described by progression and transition matrices, $\varvec{Q}^{j_0}_{i,j}$ and $\varvec{D}^{j_0}_{i,j}$ respectively, both depending on the age group at infection $j_0$. The $K \times K$ matrix $\varvec{Q}^{j_0}_{i,j}$ specifies the probabilities of moving between the undiagnosed states of the model in the ${i}$th time interval and ${j}$th age group, for individuals infected in the ${j_0}$th age group. Its ${(k,l)}$th entry is expressed as:

2 $$\begin{aligned} \left( \varvec{Q}^{j_0}_{i,j}\right) _{k,l}= \left\{ \begin{array}{l l} (1-d_{k,i,j})(1-q_{k}^{j_0}) &{} \quad \text {if} \;l=k\\ (1-d_{k,i,j})q_{k}^{j_0} &{} \quad \text {if} \; l=k+1 \text { and } k < K \\ 0 &{} \quad \text {elsewhere}\\ \end{array} \right. \end{aligned}$$

The $K \times (K+1)$ matrix $\varvec{D}^{j_0}_{i,j}$ has the ${(k,l)}$th entry giving the corresponding probability of moving from the undiagnosed state *k* to the diagnosed states $K+l$:

3 $$\begin{aligned} \left( \varvec{D}^{j_0}_{i,j}\right) _{k,l}= \left\{ \begin{array}{l l} d_{k,i,j} &{} \quad \text {if} \;l=k\\ (1-d_{k,i,j})q_{k}^{j_0} &{} \quad \text {if} \; l=K+1 \text { and } k=K \\ 0 &{} \quad \text {elsewhere}\\ \end{array} \right. \end{aligned}$$

Note that the dynamics are slightly different for individuals in state *K* as progression to AIDS is assumed to always result in a diagnosis. The above matrices reflect the assumption that the time intervals are sufficiently small so that at most one transition event can happen and that diagnosis events occur before progression. We further assume that the time intervals and age groups are of equal width.

Let $\varvec{e}_{i,j}^{j_0} = (e_{i,j,1}^{j_0}, \dots , e_{i,j,K}^{j_0})^T$ denote the $K \times 1$ vector of the expected number of individuals in the undiagnosed states in the *i*th time interval and ${j}$th age group who were infected in the $j_0$th age group. Similarly, $\varvec{\mu }_{i,j}^{j_0}= (\mu _{i,j,1}^{j_0}, \dots , \mu _{i,j,K+1}^{j_0})^T$ is a $(K+1) \times 1$ vector, with entries giving the corresponding expected numbers of new diagnoses in the absorbing states $K+1, \dots , 2K+1$. These are the result of the recursive equations:

4 $$\begin{aligned} \varvec{e}_{i,j}^{j_0} \; = \;&\left( \varvec{Q}^{j_0}_{i,j}\right) ^T \varvec{e}_{i-1,j-1}^{j_0} + \left( \varvec{Q}^{j_0}_{i,j}\right) ^T \varvec{e}_{i-1,j}^{j_0} \; \mathbb {1}_{j=A} \end{aligned}$$

5 $$\begin{aligned} \varvec{\mu }_{i,j}^{j_0} \; = \;&\left( \varvec{D}^{j_0}_{i,j}\right) ^T \varvec{e}_{i-1,j-1}^{j_0} + \left( \varvec{D}^{j_0}_{i,j}\right) ^T \varvec{e}_{i-1,j}^{j_0} \; \mathbb {1}_{j=A} \end{aligned}$$

for $j_0=1, \dots , A-1$, $i=2, \dots , T$, $j=j_0+1 \dots , \text {min}(j_0+i-1,A)$. $\mathbb {1}_{j=A}$ is an indicator function, equal to one if $j=A$ and zero otherwise. The starting values of the recursion, when $j = j_0$ are defined so that:

6 $$\begin{aligned} \varvec{e}_{i,j_0}^{j_0} \; = \;&\left( \varvec{Q}^{j_0}_{i,j_0}\right) ^T \varvec{e}_{i-1,j_0}^{j_0} \; \mathbb {1}_{j=A} + (h_{i,j_0}, 0, \dots , 0)^T \end{aligned}$$

7 $$\begin{aligned} \varvec{\mu }_{i,j}^{j_0} \; = \;&\left( \varvec{D}^{j_0}_{i,j_0}\right) ^T \varvec{e}_{i-1,j_0}^{j_0} \; \mathbb {1}_{j=A} \end{aligned}$$

for $j_0=1, \dots , A$, $i=2, \dots , T$ and we further let $\varvec{e}_{1,j_0}^{j_0} = (h_{1,j_0}, 0, \dots , 0)^T$ for $j_0=1, \dots , A$. Note that although the ${A}$th age-group, in accordance with the data is cumulative, it is assumed that infections do not occur at ages greater than $a_{A}$.

From (4) to (7), the expected number of undiagnosed individuals $\varvec{e}_{i,j}$ and the expected number of new diagnoses $\varvec{\mu }_{i,j}$ in the ${i}$th time interval and ${j}$th age group are obtained by summing over the infection age-groups $j_0$:

8 $$\begin{aligned} \varvec{e}_{i,j} \; = \;&\sum _{j_0 = \text {max}(1, j-i+1)}^j \varvec{e}_{i,j}^{j_0} \end{aligned}$$

9 $$\begin{aligned} \varvec{\mu }_{i,j} \; = \;&\sum _{j_0 = \text {max}(1, j-i+1)}^j \varvec{\mu }_{i,j}^{j_0} \end{aligned}$$

Note that the dynamic equations discussed here can be appropriately modified when, as it may happen in practice, data are available on a coarser time scale, or uneven time and age scales or they might not be collected from the beginning of the epidemic (see Sect. 1 of the online resource).

### Likelihood

The aim is to estimate the expected number of new time- and age-specific infections ${\varvec{\mathcal {H}}} = \{h_{1,1}, \dots , h_{T,A}\}$, to which we refer as the *incidence surface* (or simply *incidence*), and the diagnosis probabilities ${\varvec{\mathcal {D}}}=\{\varvec{d}_{1,1}, \dots , \varvec{d}_{T,A}\}$, when the progression probabilities ${\varvec{\mathcal {Q}}}=\{\varvec{q}^{1}, \dots , \varvec{q}^{A}\}$ are assumed to be known from external cohort studies. The components of ${\varvec{\mathcal {H}}}$ and ${\varvec{\mathcal {D}}}$ could be treated as free parameters, however a more parsimonious parameterisation can be achieved by introducing parameters $\varvec{\theta }$ and $\varvec{\delta }$ respectively, so that ${\varvec{\mathcal {H}}}\equiv {\varvec{\mathcal {H}}}(\varvec{\theta })$ and ${\varvec{\mathcal {D}}} \equiv {\varvec{\mathcal {D}}}(\varvec{\delta })$. Note that all the quantities defined in Sect. 3.2 become dependent on these parameters. For notational convenience, this dependency will be suppressed, e.g. $\varvec{d}_{i,j} \equiv \varvec{d}_{i,j}(\varvec{\delta })$, $\varvec{Q}^{j_0}_{i,j} \equiv \varvec{Q}^{j_0}_{i,j}(\varvec{\delta })$, $\varvec{D}^{j_0}_{i,j} \equiv \varvec{D}^{j_0}_{i,j}(\varvec{\delta })$, $\varvec{e_{i,j}} \equiv \varvec{e_{i,j}}(\varvec{\theta },\varvec{\delta })$, $\varvec{\mu _{i,j}} \equiv \varvec{\mu _{i,j}}(\varvec{\theta },\varvec{\delta })$, for all $i,j,j_0$.

By the properties of the non-homogeneous Poisson process (Cox and Isham 1980) characterising the infection process, the number of arrivals into the diagnosis state *k* in $(t_{i-1},t_i]$ and $(a_{j-1},a_j]$ results in a set of independent Poisson random variables with means $\mu _{i,j,k}$ [Eq. (9)]. Hence the likelihood of HIV and AIDS diagnoses is given by independent Poisson random variables, $Y^H_{i,j}$ and $Y^A_{i,j}$:

10 $$\begin{aligned} Y^A_{i,j}&\sim Po\left( \mu ^{A}_{i,j} \right) \end{aligned}$$

11 $$\begin{aligned} Y^H_{i,j}&\sim Po\left( \mu ^{H}_{i,j} \right) \end{aligned}$$

for $i= 1, \dots , T$ and $j= 1, \dots , A$, where the means are $\mu ^{H}_{i,j} = \sum _{k=1}^K \mu _{i,j,k}$ and $\mu ^{A}_{i,j} = \mu _{i,j,2K+1}$.

The contribution of the subsample of HIV diagnoses with a linked CD4 count is included based on the assumption that the distribution of the available CD4 counts is representative of the CD4 count distribution for all individuals. As the number of new HIV diagnoses in the *i*th time interval and *j*th age group is the sum of *K* independent Poisson random variables with means $\mu _{i,j,1}, \dots , \mu _{i,j,K}$, the distribution of the number of diagnoses in the states $\{K+1, \dots , 2K\}$ conditional on their sum is multinomial:

12 $$\begin{aligned} \varvec{Y}^{H_C}_{i,j} \sim \text {Multinomial}(n_{i,j}, \varvec{p}_{i,j}) \end{aligned}$$

where $$\varvec{p}_{i,j} =(p_{i,j,1}, \dots ,p_{i,j,K})$$ and $$p_{i,j,k} = \frac{\mu _{i,j,k}}{\mu ^{H}_{i,j}}, \;k= 1,\dots ,K$$.

The likelihood, expressed in terms of $\varvec{\theta }$ and $\varvec{\delta }$, is proportional to:

$$\begin{aligned} L({\mathbf {y}}^H,{\mathbf {y}}^A,{\mathbf {y}}^{H_C} \; | \; \varvec{\theta },\varvec{\delta })= & {} \; L({\mathbf {y}}^{H_C} \; | \; \varvec{\theta },\varvec{\delta }) \; L({\mathbf {y}}^H, {\mathbf {y}}^A \; | \; \varvec{\theta },\varvec{\delta })\\\propto & {} \prod _{i=1}^{T} \prod _{j=1}^{A} \left( \prod _{k=1}^{K} \left( p_{i,j,k}\right) ^{y^{H_C}_{i,j,k}} \right) e^{-\mu ^{A}_{i,j}}\left( {\mu ^{A}_{i,j}}\right) ^{y^A_{i,j}} e^{-\mu ^{H}_{i,j}}\left( {\mu ^{H}_{i,j}}\right) ^{y^H_{i,j}} \end{aligned}$$

## Bivariate smoothing methods

### Bivariate splines

To parameterise ${\varvec{\mathcal {H}}}(\varvec{\theta })$ we employ bivariate splines. In general terms, given a vector of *n* observations $\varvec{y}=(y_1, \dots , y_n)^T$ with associated two dimensional covariates $\varvec{x}=\{\varvec{x}_1, \dots , \varvec{x}_n\}$, such that $\varvec{x}_i=(x_{i 1},x_{i 2})^T$, a bivariate spline is a flexible function $g(\varvec{x}):{\mathbb {R}}^2 \rightarrow {\mathbb {R}}$ used to smoothly model the ($\varvec{x}$, $\varvec{y}$) relationship. Splines are constructed from a set of basis functions $\{b_1(\varvec{x}_i), \cdots , b_p(\varvec{x}_i)\}$ and a related $p \times 1$ vector of parameters $\varvec{\theta }$. For any $\varvec{x}$, the spline takes values $g(\varvec{x}) = \sum _j\theta _j b_j(\varvec{x})$ and can be expressed as a generalised linear model, where the data arise from a distribution of the exponential family, and with $n \times p$ design matrix $\varvec{X}$, with ${(i,j)}$th entry $\varvec{X}_{i,j}=b_{j}(\varvec{x}_{i})$. Estimation of $\varvec{\theta }$ is typically carried out through minimisation of a penalised log-likelihood criteria:

13 $$\begin{aligned} l(\varvec{\theta } | \varvec{y}) - \frac{1}{2} \sum _{s=1}^{N_s}\lambda _s \varvec{\theta ^TS}_s\varvec{\theta } \end{aligned}$$

where $l(\varvec{\theta } | \varvec{y})$ is the log-likelihood of the data and $N_s$ is the number of $p \times p$ matrices $\varvec{S_s}$ chosen to penalise the roughness of the resulting spline curve. A large number of parameters can be specified to guarantee flexibility, with any induced overfitting effect counteracted by the scaling, through the smoothing parameters $\lambda _s$, of the penalty term. Large $\lambda _s$ values favour smooth curves over more volatile ones. Closed form, and numerical, solutions are available for obtaining optimal $\varvec{{\hat{\theta }}}$ and ${\hat{\lambda }}_s$ if $\varvec{y}$ arise from a distribution from the exponential family and can be expressed as a GLM (Wood 2006a).

Note that (13) can be re-interpreted from a Bayesian perspective, as a sum of the log-likelihood and a log-prior giving a log-posterior distribution. Specifically, the penalty term is equivalent to a zero-mean multivariate Normal prior for $\varvec{\theta }$, with $p \times p$ precision matrix $\sum _{s=1}^{N_s} \lambda _s \varvec{S_s}$. Flat priors are implicitly assigned to the $\lambda _{s}$, though alternatives could be chosen.

Table 1 summarizes all the splines considered in what follows. Two main types of bivariate splines exist: thin plate splines and tensor product splines. Thin plate splines (Green and Silverman 1994) are defined by a bivariate spline basis obtained by introducing a set of knot points $\varvec{\kappa } = \{\varvec{\kappa }_1, \cdots , \varvec{\kappa }_p\}$ (see *tps* in Table 1 as well as Sect. 2.2.2 of the online resource). Roughness is quantified by the Laplacian integral:14that imposes isotropic smoothing (i.e. equal smoothing in the $x_1$ and $x_2$ dimensions) and can be conveniently expressed in a quadratic form $\varvec{\theta ^TS_1\theta }$ (in this case $N_p = 1$, see Sect. 2.1.1 of the online resource for details). Thin plate splines may be sensitive to the choice of $\varvec{\kappa }$, hence Wood (2003) proposed thin plate regression splines that avoid specifying the location of the knots (see Sect. 2.1.2, and 2.2.3 of the online resource for further details). Here a slight modification, due to Marra and Wood (2011), is implemented (see *tprs* in Table 1 as well as Sect. 2.1.3 and 2.2.4 of the online resource).

**Table 1:** A summary of the splines employed in terms of the design matrices and roughness measures

Spline	Thin plate	Thin plate regression	Tensor product thin plate regression	Tensor product B-spline
Abbreviation	*tps*	*tprs*	*ptenstprs*	*ptensbs*
$\varvec{X}$	Based on Euclidean distance between $\varvec{\kappa }$ and $\varvec{x}$	Based on eigen-decomposition of a *tps* with a knot per observation	NA	NA
$\varvec{X_{(1)}}$	NA	NA	Univariate version of *tprs*	Cubic B-spline
$\varvec{X_{(2)}}$	NA	NA	Univariate version of *tprs*	Cubic B-spline
$\varvec{S}$	Eq. 14	Based on eigen-decomposition of Eq. 14	NA	NA
$\varvec{S_{(1)}}$	NA	NA	Univariate version of Eq. 14	First order difference squared
$\varvec{S_{(2)}}$	NA	NA	Univariate version of Eq. 14	Second order difference squared
Online Resource	2.2.2	2.2.3–4	2.2.5 and 2.1.3	2.2.5 and 2.1.4

For tensor product splines, the spline basis is a product of two univariate splines in the time and age dimensions denoted by subscripts (1) and (2)

Tensor product splines are constructed by defining two univariate splines, with design matrices $\varvec{X_{(1)}}$ and $\varvec{X_{(2)}}$, (of dimension $n_1 \times p_1$, and $n_2 \times p_2$) and roughness matrices $\varvec{S_{(1)}}$ and $\varvec{S_{(2)}}$ (of dimension $p_1 \times p_1$ and $p_2 \times p_2$). The bases for the joint bivariate spline are then obtained by multiplying the basis functions of the marginal splines. Tensor product splines allow for differential smoothing in the two dimensions ($N_p=2$), by applying the univariate penalty matrices marginally (see Sect. 2.2.5 of the online resource). Eilers and Marx (2003) constructed tensor product splines from marginal cubic B-spline and measuring marginal roughness via a first order difference penalty squared (see *ptensbs* in Table 1). Wood (2006b) extended their approach to handle any type of marginal spline, such as thin plate regression splines, and any type of marginal penalty, such as the integrated second derivative squared (see *ptenstprs* in Table 1). The different penalty measures of *ptensbs* and *ptenstprs* imply that, in absence of information, the marginal splines revert towards a flat and linear trend respectively.

### Splines within back-calculation

The bivariate splines are used to model the log-incidence surface $\varvec{\gamma } = (\gamma _{1,1}, \dots , \gamma _{T,A})^T$, where $\gamma _{i,j} = log(h_{i,j})$, by letting $\varvec{\gamma } = \varvec{X} \varvec{\theta }$. $\varvec{X}$ denotes the design matrix corresponding to the chosen type of spline. Irrespectively of the parameterisation of ${\varvec{\mathcal {D}}}(\varvec{\delta })$, back-calculation cannot be expressed as a GLM: the likelihood (Eq. 13) includes Poisson and Multinomial terms so that a single link function cannot be specified, and the expected number of diagnoses $\varvec{\mu _{i,j}}$ is a non-linear function of $\varvec{\theta }$ and $\varvec{\delta }$. In this case, standard algorithms to estimate the spline parameters $\varvec{\theta }$ and $\lambda _s$ within a GLM penalised likelihood context cannot be implemented. Although the penalised likelihood can be numerically maximized, estimation of $\lambda _s$ and quantification of uncertainty become computationally prohibitive (Brizzi 2018, Sect. 6.4.4). Even Expectation Maximization (EM) based algorithms, often used for back-calculation (Becker et al. 1991; Becker and Marschner 1993; Marschner and Bosch 1998) cannot be efficiently employed, as the derivatives of the likelihood are not analytically tractable.

An alternative Bayesian approach (Wood 2016) offers a number of advantages allowing direct estimation of both model and smoothing parameters and automatic quantification of uncertainty. Moreover external sources of information (e.g. under-reporting rates, see Birrell et al. 2012) can easily be incorporated and implementation can be achieved using standard software for Bayesian analysis.

## Simulation study

Here we investigate the most appropriate type of spline model. To do this, we carried out a simulation study starting from the age-dependent back-calculation model described in Sect. 3.1 with $K=4$ undiagnosed states, defined by CD4 count classes: $[500,\infty )$, [350, 500), (200, 350] and (0, 200]. Using yearly time steps to define both the time intervals and age groups (see Sect. 1.2 of the online resource), 20 time intervals and 52 age groups are considered, corresponding to ages 15 ($j=1$) to 66 ($j=52$). The starting point is taken to be an intermediate point in the history of the epidemic and the expected number of undiagnosed infections (by state) $\varvec{\pi }^\star$ is specified (see Sect. 1.1 of the online resource). Values for the data-generating incidence surface ${\varvec{\mathcal {H}}}^\star = \{h_{1,1}^\star , \dots , h^\star _{20,52}\}$, diagnosis probabilities ${\varvec{\mathcal {D}}}^\star =\{d^\star _{1,1,1}, \dots , d^\star _{4,20,52}\}$, progression probabilities ${\varvec{\mathcal {Q}}}^\star =\{q^\star _{1}, \dots , q^\star _{52}\}$, and $\varvec{\pi }^\star$ were chosen to reflect realistic values for the MSM-HIV epidemic in England between 1995 and 2015 based on previous studies (Aalen et al. 1997; Sweeting et al. 2005; Birrell et al. 2012). The data-generating expected number of annual HIV diagnoses, AIDS diagnoses and CD4 proportions, denoted $\mu ^{H\star }_{i,j}$, $\mu ^{A\star }_{i,j}$ and $\varvec{p}^\star _{i,j}$ respectively, are then obtained through a generalisation of the dynamical equations described in (5) and (9) (see Sect. 3.1 in the online resource). These are used to simulate data according to (10–12), taking $n^\star _{i,j}$ to be equal to the $n_{i,j}$, i.e. the number of samples observed in the last 20 years of this study (Fig. 1a).

**Fig. 4:**
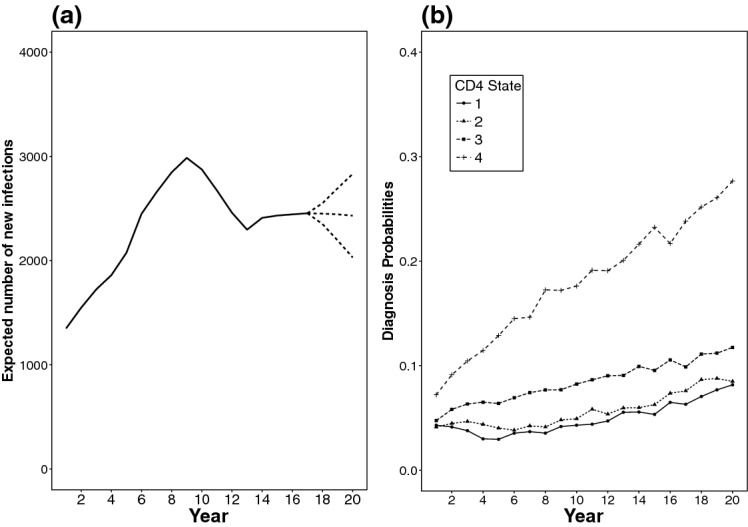
**a** Time profiles of the three incidence surfaces used for data generation. The dashed lines denote the increasing, flat and decreasing scenarios for incidence in most recent years. **b** Diagnosis probabilities used for data generation, by undiagnosed state

**Fig. 5:**
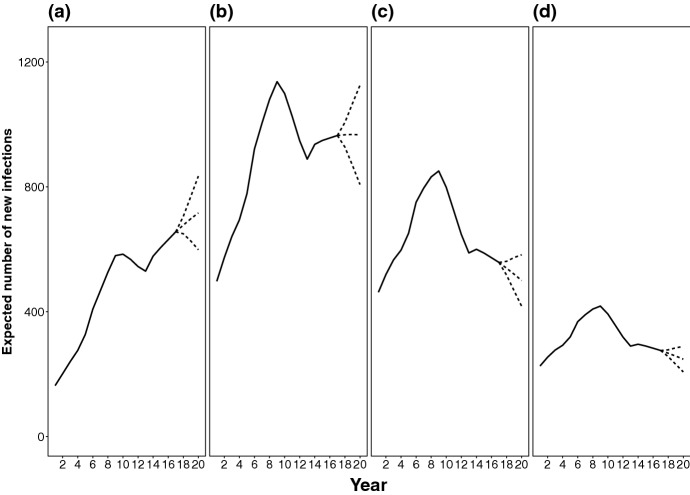
Time profiles of the three data-generating incidence surfaces, stratified by age-range: **a** 15–24; **b** 25–34, **c** 35–44, **d** 45+

Three data-generating bivariate incidence surfaces are derived by assuming: $h_{i,j} = h_i v_{i,j}$, where $h_i$ is the total number of expected infections in $(t_{i-1},t_i]$ and $v_{i,j}$ is the proportion of $h_i$ occurring among age groups $(a_{j-1},a_j]$, with $\sum _{j=1}^{52} v_{i,j}=1$, for all *i*. Three plausible time profiles $h_i$ are considered. These are identical until the most recent 3 years when they differ to allow an increasing, a constant and a decreasing trend in incidence (see Fig. 4a). The $v_{i,j}$ are constructed such that, in all the three time profiles, the mean age at infection shifts linearly from age 43, in $(t_0,t_1]$, to 33, in $(t_{19},t_{20}]$. The resulting age-specific time profiles of the incidence surfaces are shown in Fig. 5.

To limit the computational burden of the simulation study, the diagnosis probabilities used to generate the data (Fig. 4b) are assumed to be independent of age, i.e. $d_{k,i,j} \equiv d_{k,i}$ for all *j*. The values specified are available in the online resource, Sect. 3.

### Study design

For each of the three incidence surfaces considered, 50 sets of simulated data were generated. Estimation of the incidence surface was then carried out for each simulated dataset, with the incidence surface modelled using each of the four splines discussed in Table 1. All splines considered have 80 parameters. For thin plate splines, knots are located at intervals of 2 years in the time dimension, and every 6.5 years in the age dimension (i.e. for a total of 10 and 8 knots in the time and age dimension respectively). For each of the two marginal splines of a tensor product we specified 10 and 8 parameters in the time and age dimension respectively (for a total of 80 parameters), using equidistant knots. The weakly informative priors imposed on the reparameterised coefficients are available in Sect. 3.5 of the online resource.

The smoothing parameters $\lambda _s$ have a crucial role as they determine the roughness of the estimated incidence curve. To reflect a lack of prior knowledge and a weak preference towards smooth curves, diffuse half-t prior distributions with 2 degrees of freedom and scale parameter 200 are chosen so that 95% of the prior density lies in the [0, 400] region (Gelman 2006).

Alongside the smoothed infection process, a model for the diagnosis process ${\varvec{\mathcal {D}}}(\varvec{\delta })$ is also specified. Diagnosis probabilities are expressed on a logistic scale, i.e. $\delta _{k,i} = \log \left( \frac{d_{k,i}}{1-d_{k,i}}\right)$, using a first order random walk:

15 $$\begin{aligned} \delta _{k,i} \sim N(\delta _{k,i-1}, \sigma ^2_{k}), \quad i= 2, \dots , 20, \; k= 1, \dots , 4 \end{aligned}$$

A total of 600 scenarios are considered, where each scenario refers to a combination of the data-generating incidence surface, a spline model for $\varvec{\gamma }$ (e.g. *tprs*) and a simulated dataset.

Inference is carried out using Stan (version 2.14), which employs Hamiltonian Monte Carlo methods (Hoffman and Gelman 2014; Carpenter et al. 2017). Each posterior estimate is obtained using three chains of 2000 iterations with burn-in of 1000. Splines are implemented via the R package **mgcv** (Wood 2017), and the reparameterisations discussed in Wood (2016) are implemented for computational efficiency (see online resource, Sect. 2.3). The weakly informative priors imposed on the reparameterised spline coefficients, $\delta _{k,1}$ and $\sigma ^2_{k}$ parameters are available in Sect. 3.5 of the online resource. The approximate running time per scenario is 10 h. Codes are available at https://github.com/frbrz25/Thesis_Codes.

### Assessment

For the ${m}$th $(m=1, \dots , 600)$ scenario, posterior distributions for the incidence surface and the diagnosis probabilities for each diagnosis state are obtained with ${\varvec{\mathcal {{\widehat{H}}}}}^m= \{{\widehat{h}}_{1,1}^m, \dots , {\widehat{h}}_{1,A}^m, \dots , {\widehat{h}}_{T,1}^m, \dots {\widehat{h}}_{T,A}^m\}$ and ${\varvec{\mathcal {{\widehat{D}}}}}_k^m = \{{\widehat{d}}_{k,1}^m, \dots , {\widehat{d}}_{k,T}^m\}$ denoting the corresponding pointwise posterior means respectively. The corresponding $\alpha /2$ quantiles of the posterior distributions are denoted ${\varvec{\mathcal {{\widehat{H}}}}}^{m, \alpha /2} = \{{\widehat{h}}_{1,1}^{m,\alpha /2}, \dots , {\widehat{h}}_{T,A}^{m,\alpha /2}\}$ and ${\varvec{\mathcal {{\widehat{D}}}}}_k^{m,\alpha /2} = \{{\widehat{d}}_{k,1}^{m,\alpha /2}, \dots , {\widehat{d}}_{k,T}^{m,\alpha /2}\}$.

The Predictive Mean Squared Error (PMSE) is the mean of squared errors between the data-generating and the estimated incidence curves. For the *m*th scenario this has the expression:

16 $$\begin{aligned} \text {PMSE}({\varvec{\mathcal {{\widehat{H}}}}}^m) = \frac{1}{TA}\sum _{i=1}^{T} \sum _{j=1}^A \left( {\widehat{h}}^m_{i,j} - h^\star _{i,j}\right) ^2 \end{aligned}$$

The distribution of $\text {PMSE}({\varvec{\mathcal {{\widehat{H}}}}}^m)$ can be evaluated for different splines, with lower $\text {PMSE}({\varvec{\mathcal {{\widehat{H}}}}}^m)$ values indicating that the data-generating incidence curve is more accurately estimated. $\text {PMSE}({\varvec{\mathcal {{\widehat{D}}}}}_k^m)$ for the diagnosis probabilities, from the *k*th state can analogously be defined.

Convergence of HMC chains of 1000 (post burn-in) iterations is assessed using the ${\hat{R}}$ statistics of Gelman and Rubin (1992).

**Fig. 6:**
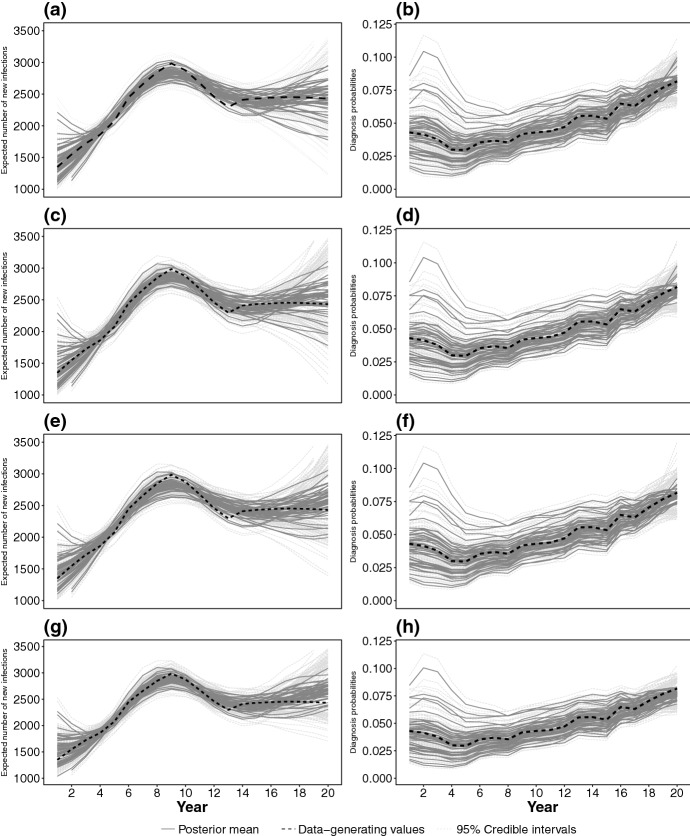
Pointwise posterior mean (gray solid lines) and 95% credible intervals (light gray dotted lines) of the incidence surface time profile (**a**, **c**, **e**, **g**) and diagnosis probabilities from state 1 (**b**, **d**, **f**, **h**) for the different splines: *tps* (**a**, **b**); *tprs* (**c**, **d**); *ptenstprs* (**e**, **f**); *ptensbs* (**g**, **h**). Black dashed lines represent the values used in data generation

**Fig. 7:**
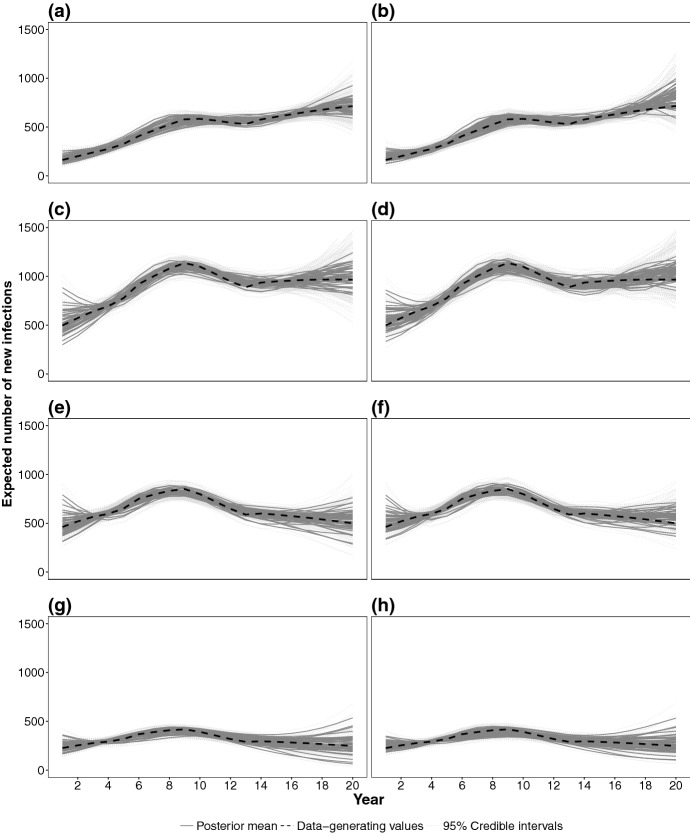
Pointwise posterior mean (gray solid lines) and 95% credible intervals (light gray dotted lines) of the incidence surface time profile for the *tprs* (**a**, **c**, **e**, **g**) and *ptensbs* (**b**, **d**, **f**, **h**), stratified by age ranges: 15–24 (**a**, **b**), 25–34 (**c**, **d**), 35–44 (**e**, **f**), and 45+ (**g**, **h**). Black dashed lines represent the values used in data generation

**Fig. 8:**
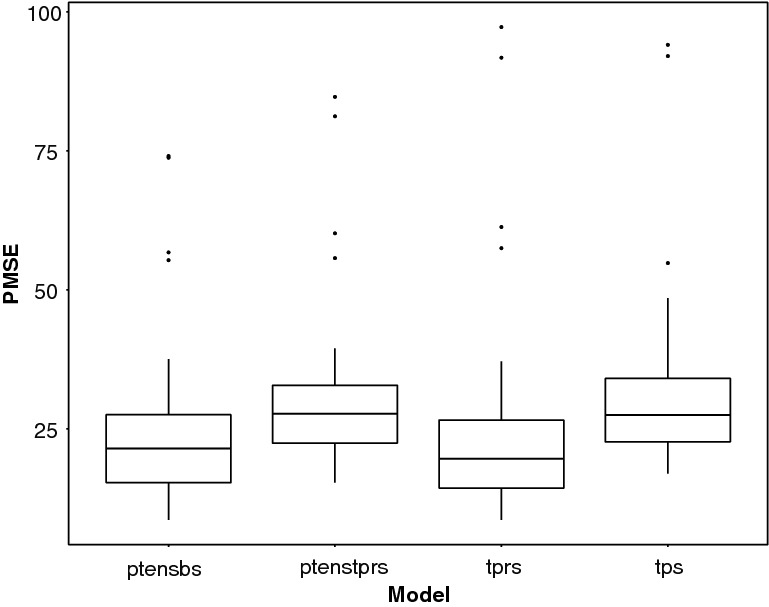
Comparison of the performance of four spline models in terms of the distribution of the predictive mean square error PMSE

### Results

Figure 6 shows that all the spline models considered (*tps*, *tprs*, *ptenstprs*, *ptensbs*) reasonably reproduce the time profile of the flat incidence surface, except in the first and last 3 years of the epidemic, where estimates diverge. Estimates in the initial period are sensitive to the choice of the initial expected number of infected individuals $\varvec{\pi }^\star$. A further sensitivity analysis (see Brizzi 2018) showed that these estimates are only affected by $\varvec{\pi }^\star$ for, at most, a period of 7 years.

In the last 3 years, the time profiles of the incidence surface are overestimated in the majority of the scenarios under each spline model (especially *ptenstprs* and *ptensbs*). This is induced by an incorrect attribution of recent diagnoses to an increase in incidence (Fig. 6) resulting in a consistent under-estimation of the diagnosis probabilities from state 1 in most recent years. There is also increased variability across the estimates at this time, a common feature of back-calculation.

The age-specific time profiles of the incidence are adequately estimated for all age ranges and spline models. In Fig. 7 aggregated incidence over the 15–24 and 25–34 age ranges are accurate, even in the later years. Estimates in the 35–44 and 45+ age-ranges are more volatile, due to fewer diagnoses occurring in these age-groups.

Figure 8 shows $\text {PMSE}({\varvec{\mathcal {{\widehat{H}}}}}^m)$ for each of the splines. Among thin plate splines, *tprs* outperform *tps* (similar findings were obtained in Wood 2003). Among tensor product splines, *ptensbs* outperforms *ptenstprs* with the $\text {PMSE}({\varvec{\mathcal {{\widehat{H}}}}}^m)$ distributions of the *tprs* and *ptensbs* being similar. The incidence time profiles, estimated by *tprs* and *ptensbs* (Fig. 7), only differ in the latest years, with the estimates from *tprs* visibly less biased, but more volatile (especially in most recent years). This different performance is attributable to the assumptions on the behaviour of the splines in most recent years, for which data are only weakly informative. The linear trend of the *tprs* splines, occasionally results in extreme estimates, whereas the *ptensbs* flattens out.

In this simulation study, *tprs* and *ptensbs* splines perform similarly. Note that the time intervals and age groups are both measured on a yearly scale and hence the isotropy assumption appears to hold. This assumption is hardly testable in practice and does not apply in situations where data are collected on an uneven time and age scale; as *ptensbs* splines do not rely on isotropy, they may be preferred to *tprs* splines. All of the above conclusions consistently apply when also considering the increasing and decreasing incidence profiles for recent infections.

Additionally there was no detectable difference in the goodness-of-fit achieved by the different spline incidence models (see Brizzi 2018, Appendix G.2.1.).

A further sensitivity analysis (see Brizzi 2018, Sect. 4.6.4) revealed that incidence estimates are robust to the specified weakly informative prior for the smoothing parameters $\lambda _s$.

## Application to the MSM-HIV epidemic in England and Wales

As an illustration, we apply the back-calculation model described in Sect. 5, to the data introduced in Sect. 2. Specifically, we focus on reconstructing incidence from the mid 1990s when CD4 data started to become more reliable, including a total of 45,972 diagnoses from 1995 to 2015. Individuals are assumed to have seroconverted between 15 and 66 years of age. The expected number of undiagnosed individuals at the beginning of 1995 (i.e. $\varvec{\pi }$) and progression probabilities ${\varvec{\mathcal {Q}}}$ are set as in Sects. 3.2 and 3.3 of the online resource.

### Initial investigations

As in Sect. 5 a yearly scale for both time and age (i.e. $T=21$, $A=52$) is assumed. Incidence is modelled using a *ptensbs* spline and diagnosis probabilities through a random walk on a logistic scale, independent of current age. As before, models are implemented in Stan, using four chains of 2000 iterations, the first 1000 of which were burn-in. The resulting posterior sample of 4000 iterations was obtained in approximately 8 h.

**Fig. 9:**
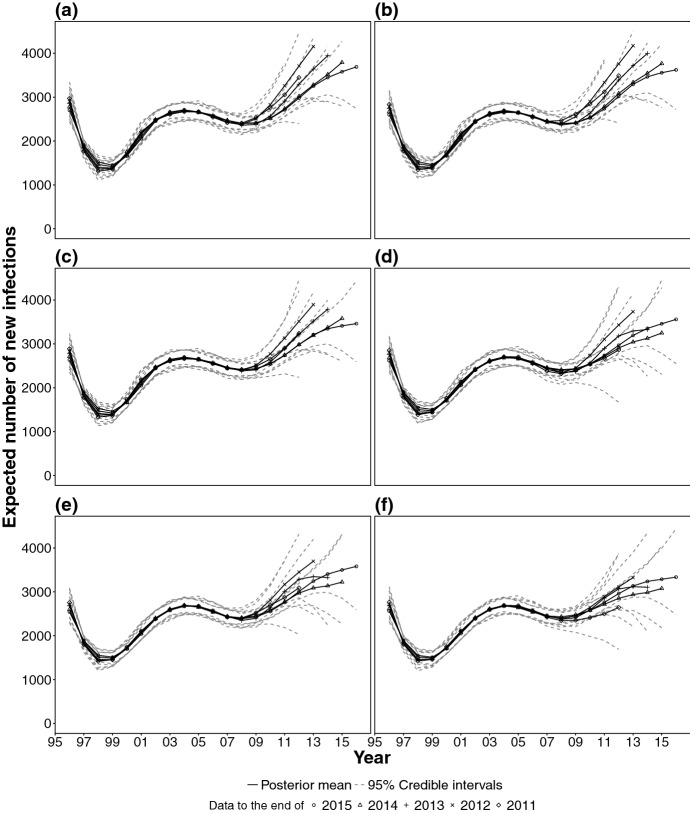
Posterior mean of the incidence surface time profile, estimated using data up to the end of 2011, 2012, 2013, 2014, and 2015, stratified by model: **a***YAID*; **b***QAID*; **c***YADD0*; **d***QADD0*; **e***YAAD1*; **f***QAAD1*

Figure 9a is a plot of the estimated incidence surfaces obtained by sequentially including an additional year of data from 2010 to 2015. Let ${\hat{h}}_{i}^{y}$ denote the estimate of the time profile of incidence in the *i*th year, using data up to the end of the *y*th year, i.e. ${\hat{h}}_{i}^{y} = \sum _{j=1}^A {\hat{h}}_{i,j}^{y}$. Note that ${\hat{h}}_{12}^{12}$ and ${\hat{h}}_{13}^{13}$ are approximately 4000, but are revised downwards (i.e. ${\hat{h}}_{12}^{15}$ and ${\hat{h}}_{13}^{15}$) to approximately 2500 when data up to the end of 2015 are used. The additional 2 years of data are informative about infection levels in 2012 and 2013 and thus the increasing trend estimated using data up to the end of 2012 and 2013 is potentially misleading.

### Investigating the robustness of the model

Ensuring the robustness of the model in most recent years is crucial. Additional flexibility, achieved by considering a finer (quarterly) time scale and/or extending the model to make the diagnosis probabilities dependent on current age, may allow the model to better adapt to recent changes in the data.

We consider three models for the age-dependence of diagnosis probabilities and two alternative time scales, using six models in total.

1. *YAID*: yearly model, with age-independent diagnosis probabilities (as discussed in the previous section). Let $i= 2, \dots , T$, $j=1, \dots , A$, $k=1, \dots , 4$ and:
  17 $$\begin{aligned} \delta _{k,i,j} = \delta _{k,i-1,j} + \sigma _k\epsilon _{ik}, \qquad \epsilon _{ik} \sim N(0, 1) \end{aligned}$$
  with initial condition:
  18 $$\begin{aligned} \delta _{k,1,j} = m_k + \sigma _{0,k} \epsilon _{1k}, \qquad \epsilon _{1k} \sim N(0, 1) \end{aligned}$$
  where $m_k$ and $\sigma _{0,k}$ are known fixed constants, whereas $\sigma _k$ are estimated.
2. *YADD0*: As *YAID*, except for an additive term $\alpha _{j}$ in (17) and (18). This term has the interpretation of an age-specific linear time trend in the logistic diagnosis probabilities and $\alpha _{j}$ is estimated, after imposing a N(0, 1) prior on it.
3. *YADD1*: As *YADD0*, except that we use an age and state specific $\alpha _{j,k}$ time trend in the logistic diagnosis probabilities.
4. *QAID*: As *YAID*, but using a quarterly time scale.
5. *QADD0*: As *YADD0*, but using a quarterly time scale.
6. *QADD1*: As *YADD1*, but using a quarterly time scale.

Figure 9 displays the sensitivity of the estimated time profile of the incidence surface to the sequential addition of further years of data and demonstrates that the *QAAD1* model is the most robust among the models considered. Estimates of incidence, both at population and at age-specific level in the most recent years are only slightly revised when further years of data are added, suggesting that the estimated trends in incidence are not artificial.

All the models are consistent with the HIV diagnosis data, as judged by the goodness of fit to the observed data (Brizzi 2018, Appendix H.4.). However the *YAAD1* and *QAAD1* seem to better fit the AIDS and CD4 count data, especially in the 15–24 and 45+ age ranges. The number of diagnoses with CD4 count in the (200, 500] range, between 2005 and 2015, only increase in the 15–24 age range. A model with a state-and-age dependent starting value for the diagnosis probabilities, allows this feature of the data to be captured. For all age ranges, the posterior-predictive distribution of CD4 count data include all data points, but credible intervals are wide. Although overfitting may be an issue, as suggested by the noisy fit to CD4 data, *QAAD1* successfully achieves robust incidence estimates.

**Fig. 10:**
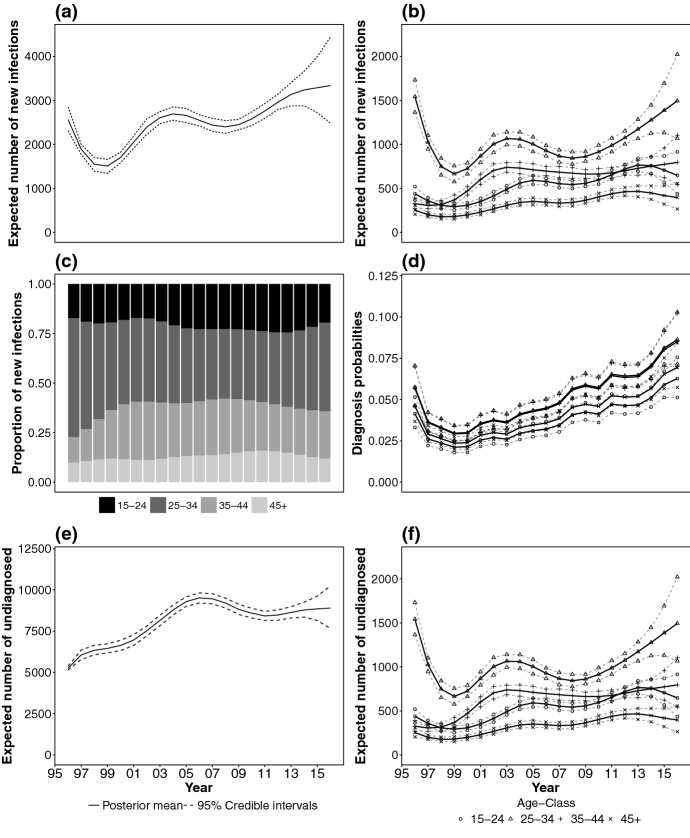
Posterior mean (and related 95 % credible intervals) of a number of relevant quantities for the chosen final model, *QAAD1*: **a** incidence surface time profile; **b** incidence surface time profile, stratified by age range; **c** proportion of incidence in each age range over time; **d** diagnosis probabilities from state 1; **e** expected number of undiagnosed individuals; **f** expected number of undiagnosed individuals, by age range

### Illustration results

Figure 10 shows the results obtained from the *QAAD1* model. Figure 10a plots the expected number of infections over time; incidence has steadily increased from 2007 onwards, even though a plateau is reached in the latest years. However, age-specific back-calculation reveals that this plateau hides a sharp increase in the expected number of infections for the 25–34 age-group from 2007 (Fig. 10b). Incidence has remained approximately constant in this period for the other age ranges. As a result the distribution of age at infection has shifted towards younger ages: in 2000, 17%, 42%, 30%, 11% of individuals were respectively newly infected in age ranges 15–24, 25–34, 35–44 and 45+, compared to 19%, 45%, 24%, 12% of individuals in 2015 (Fig. 10c).

Similarly, Fig. 10d shows that the diagnosis probabilities from state 1 vary with age, and are estimated to be higher for the 25–34 and the 35–44 age ranges.

The age-dependent back-calculation model further reveals that underlying a constant trend in the expected number of undiagnosed infections (Fig. 10e) in the last 5 years, there is a sharp increase in the expected number of undiagnosed individuals living with HIV in the 25–34 age range, and a sharp decrease in the 35–44 age range (Fig. 10f).

It is further interesting to note that age-dependent incidence estimates are reassuringly in agreement with results obtained from the simpler age-independent model (Fig. 2), apart from the 1995–1998 period. Over these years the incidence estimates are highly sensitive to the choice of $\varvec{\pi }^\star$, the age specific distribution of the infected undiagnosed population in 1995, which is instead estimated from historical data in the age-independent model. Results from the two models become consistent after 1998, when results are no longer influenced by the specification of $\varvec{\pi }^\star$.

## Discussion

Back-calculation plays an important role in the monitoring of HIV incidence, based on routinely collected surveillance data. The contributions of Odd Aalen to the back-calculation literature, through pioneering the exploitation of newly available sources of data (Aalen et al. 1994; Farewell et al. 1994), particularly within a multi-state model (Aalen et al. 1997; Sweeting et al. 2005), have been fundamental. These ideas have been central to the development of multi-state back-calculation, where the incorporation of information on CD4 count data around HIV diagnosis, has also enabled estimation of trends in diagnosis probabilities and, consequently, trends in the number of undiagnosed infections (Birrell et al. 2012). In this paper, we have proposed a further extension of this CD4-staged back-calculation model, which allows the joint estimation of age- and time-specific HIV incidence, as well as age- and time-specific diagnosis probabilities. This insight into the HIV epidemic is extremely valuable for targeting and evaluating interventions aimed at reducing HIV prevalence and transmission.

Existing approaches to smoothing incidence over time and age used strong multiplicative assumptions or step functions, which require the arbitrary definition of corner-points. We have thoroughly investigated spline models for smoothing incidence jointly over time and age at infection at a finer level of detail (52 yearly age groups, 80 quarterly time periods). Bivariate splines allow the capture of age- and time-interactions in a continuous manner, with tensor product splines permitting differential smoothing in the two dimensions. Results from the simulation study show that tensor product splines, constructed from marginal cubic B-splines measuring roughness with first order difference penalty squared (*ptensbs*), are particularly suitable.

Any back-calculation model provides very uncertain incidence estimates over the most recent period, which are the most crucial to inform public health decision making. This is still true, to some extent, for the model we propose here, motivating a further extension of the backcalculation to incorporate additional data on biomarkers indicative of recent infection (Ndawinz et al. 2011; Yan et al. 2011). Since 2009, PHE has introduced the routine application of Recent Infection Testing Algorithms (RITA) to new HIV diagnoses, allowing the identification of ‘recent’ infections (Aghaizu et al. 2014). In principle, the proposed multi-state back-calculation framework could be extended through the addition of undiagnosed states for newly infected individuals to include RITA data. In practice, this poses some challenges: many new diagnoses are not RITA tested; and an increase in the number of states will result in both model complexity and computational demand. The approach proposed here already requires long running times, and consideration of a reduced and/or more coarse time scale is often necessary to achieve implementation within an acceptable computational budget. Running times are of the order of 10 and 80 h for a yearly and quarterly time scale, respectively, even when only considering the last 20 years of the epidemic. Despite being faster to implement, yearly models produce estimates that are substantially less stable (e.g. to the addition of further years of data) than the respective quarterly estimates. To successfully incorporate RITA data, future research would benefit from focusing on more computationally efficient inferential approaches than used here.

We have presented a method to estimate age-specific trends in incidence, diagnosis and undiagnosed prevalence of HIV using information from routine surveillance. New diagnosis and early infection biomarker data are becoming increasingly available worldwide, even in less developed countries. Our approach is of value in those countries, as our model can be easily adapted to accommodate limited historical data. By assuming an initial distribution $\varvec{\pi }^\star$ for the infected individuals across the undiagnosed states at a convenient starting point, age-specific incidence can still be estimated with results sensitive to the choice of $\varvec{\pi }^\star$ only for the initial years. In countries with established surveillance systems as England and Wales, our approach represents an insightful new tool to guide the targeting of test and treat and pre-exposure prophylaxis strategies (Volz et al. 2018) and to support their evaluation.

## Electronic supplementary material

Below is the link to the electronic supplementary material.

Supplementary material 1 (pdf 223 KB)
