# Supplementary material for: Extending Bayesian back-calculation to estimate age and time specific HIV incidence
Source: Lifetime Data Anal. 2019 Feb 27;25(4):757–80. doi: 10.1007/s10985-019-09465-1 (PMC6776486; doi:10.1007/s10985-019-09465-1)
Supplement: Supplementary file 1 — Supplementary material 1 (pdf 223 KB) [file 10985_2019_9465_MOESM1_ESM.pdf]

Noname manuscript No.
(will be inserted by the editor)

Appendix - Extending Bayesian back-calculation to estimate age and time specific HIV incidence (Online resource, *Lifetime Data Analysis*)

Francesco Brizzi, Paul J. Birrell, Martyn T. Plummer, Peter Kirwan, Alison Brown, Valerie Delpech, Owen Noel Gill, Daniela De Angelis

the date of receipt and acceptance should be inserted later

1 Extending the dynamics of the back-calculation model

In Section 3.2 of the main paper, we mention that the dynamics of the back-calculation model can be extended to handle surveillance data which are not collected for the full epidemic time period (Section 1.1), on a coarse time scale (Section 1.2) and on different time and age-scales (Section 1.3)

1.1 Back-calculation over a reduced time period

Formulation of the back-calculation model can be considered on a subset $(t_b, t_T] \times (a_0, a_A]$ of the full epidemic period, where $t_b > t_0$ is chosen based on the availability of surveillance data or on computational constraints. This requires specification the expected number of individuals undiagnosed in the model at time t_b, stratified by undiagnosed state $\{1, \dots, K\}$, age (interval) at infection and current age (interval) at t_b. In practice, pre t_b modelling, where data may simply not be available, is required to gather information on the age at infection. We therefore assume that initially undiagnosed individuals progress according to their calendar age at t_b, rather than their age at infection, and denote initially undiagnosed individual in the jth age group by the $A \times 1$ vectors $\boldsymbol{\pi}_j = (\pi_{j,1}, \dots, \pi_{j,K})^T$, $j = \{1, \dots, A\}$.

The model dynamics, for time $(t_{b+i-1}, t_{b+i}]$ and age $(a_{j-1}, a_j]$ intervals $(i = \{1, \dots, T-b\},\ j = \{1, \dots, A\})$, can be obtained by setting $e^{j_0}_{1,j_0} = (h_{1,j_0} + \pi_{j,1}, \pi_{j,2}, \dots, \pi_{j,K})^T$ (instead of $e^{j_0}_{1,j_0} = (h_{1,j_0}, 0, \dots, 0)^T$ as in Section 3.2 of the main paper).

Address(es) of author(s) should be given

Misspecification of $\boldsymbol{\pi} = \{\boldsymbol{\pi}_1, \ldots, \boldsymbol{\pi}_A\}$ might lead to biased incidence and diagnosis probabilities estimates.

1.2 Back-calculation on a coarser time scale

Epidemic data may only be available on a coarse scale; in this situation allowing at most one transition between the model states in an interval does not allow infected individuals to be diagnosed rapidly enough. Yet the model dynamics over a coarse scale can be constructed by considering smaller subintervals, where the above assumption holds.

Coarse time intervals $(t_{i-1}, t_i]$, $i = \{1, \ldots, T\}$ and age groups $(a_{j-1}, a_j]$, $j = \{1, \ldots, A\}$ are split into N_s intervals of equal length, denoted $(t_{i,s-1}, t_{i,s}]$ and $(a_{j,s-1}, a_{j,s}]$, where $s = \{1, \ldots, N_s\}$ and $t_{i,0} \equiv t_{i-1}$, $t_{i,N_s} \equiv t_i$, $a_{j,0} \equiv a_{j-1}$, $a_{j,N_s} \equiv a_j$. At most one move between the model states is allowed in the sub-intervals, so that up to N_s events can occur in a "coarse" interval.

Let $h_{i,j_0,s}$ denote the expected number of new infections in $(t_{i,s-1}, t_{i,s}]$ and $(a_{j_0,s-1}, a_{j_0,s}]$. $d_{k,i,j,s}$ denotes the diagnosis probability in $(t_{i,s-1}, t_{i,s}]$ and $(a_{j,s-1}, a_{j,s}]$ from undiagnosed state k, whereas $q^{j_0}_{k,s}$ denotes the progression probability from undiagnosed state k for an individual infected in $(a_{j_0,s-1}, a_{j_0,s}]$. As only one movement between the model states is allowed in $(t_{i,s-1}, t_{i,s}]$ and $(a_{j,s-1}, a_{j,s}]$, the transition $(\boldsymbol{Q}^{j_0}_{i,j,s})$ and progression $(\boldsymbol{D}^{j_0}_{i,j,s})$ matrices can be defined via Equations (2) and (3) in the main paper. Moreover, all the quantities defined are constant within the N_s sub-intervals:

$$\begin{array}{ll}
h_{i,j_0} \equiv h_{i,j_0,1} = \cdots = h_{i,j_0,N_s}, & i = \{1,\ldots T\},\ j_0 = \{1,\ldots A\} \\
d_{k,i,j} \equiv d_{k,i,j,1} = \cdots = d_{k,i,j,N_s}, & i = \{1,\ldots T\},\ j = \{1,\ldots,A\},\ k = \{1,\ldots,K\} \\
q^{j_0}_{k} \equiv q^{j_0}_{k,1} = \cdots = q^{j_0}_{k,j,N_s}, & j_0 = \{1,\ldots,A\},\ k = \{1,\ldots,K-1\} \\
\boldsymbol{Q}^{j_0}_{i,j} \equiv \boldsymbol{Q}^{j_0}_{i,j,1} = \cdots = \boldsymbol{Q}^{j_0}_{i,j,N_s}, & i = \{1,\ldots T\},\ j = \{1,\ldots,A\},\ j_0 = \{1,\ldots,A\} \\
\boldsymbol{D}^{j_0}_{i,j} \equiv \boldsymbol{D}^{j_0}_{i,j,1} = \cdots = \boldsymbol{D}^{j_0}_{i,j,N_s}, & i = \{1,\ldots T\},\ j = \{1,\ldots,A\},\ j_0 = \{1,\ldots,A\}
\end{array}$$

Now the expected number of undiagnosed infections $(\boldsymbol{e}^{j_0}_{i,j})$ and new diagnoses $(\boldsymbol{\mu}^{j_0}_{i,j})$ at the end of each "coarse" i^{th} time interval and j^{th} age group, for infection occurring in the j_0^{th} age group, can be expressed using the following recursive equations, for $j_0 = \{1, \ldots, A-1\}$, $i = \{2, \ldots, T\}$, $j = \{j_0 + 1 \ldots, min(j_0 + i - 1, A)\}$:

$$\boldsymbol{e}^{j_0}_{i,j} = \left(\widetilde{\boldsymbol{Q}}^{j_0}_{i,j}\right)^{\boldsymbol{T}} \boldsymbol{e}^{j_0}_{i-1,j-1} + \left(\widetilde{\boldsymbol{Q}}^{j_0}_{i,j}\right)^{\boldsymbol{T}} \boldsymbol{e}^{j_0}_{i-1,j}\ \delta_{j,A} \tag{1}$$

$$\boldsymbol{\mu}^{j_0}_{i,j} = \left(\widetilde{\boldsymbol{D}}^{j_0}_{i,j}\right)^{\boldsymbol{T}} \boldsymbol{e}^{j_0}_{i-1,j-1} + \left(\widetilde{\boldsymbol{D}}^{j_0}_{i,j}\right)^{\boldsymbol{T}} \boldsymbol{e}^{j_0}_{i-1,j}\ \delta_{j,A} \tag{2}$$

where:

$$\widetilde{\boldsymbol{Q}}^{j_0}_{i,j} = \left(\boldsymbol{Q}^{j_0}_{i,j}\right)^{N_s} \qquad \widetilde{\boldsymbol{D}}^{j_0}_{i,j} = \sum_{s=0}^{N_s-1} \left(\boldsymbol{Q}^{j_0}_{i,j}\right)^{s} \boldsymbol{D}^{j_0}_{i,j} \tag{3}$$

The initial values of the recursion are for $j_0 = \{1, \ldots, A\}$, $i = \{2, \ldots, T\}$:

$$\boldsymbol{e}^{\boldsymbol{j_0}}_{\boldsymbol{i,j_0}} = \left(\sum_{s=0}^{N_s-1} \left(\boldsymbol{Q}^{\boldsymbol{j_0}}_{\boldsymbol{i,j_0}} \right)^s \right)^T \boldsymbol{h}_{\boldsymbol{i,j_0}} + \left(\widetilde{\boldsymbol{Q}}^{\boldsymbol{j_0}}_{\boldsymbol{i,j_0}} \right)^{\boldsymbol{T}} \boldsymbol{e}^{\boldsymbol{j_0}}_{\boldsymbol{i-1,j_0}} \, \delta_{j,A} \tag{4}$$

$$\boldsymbol{\mu}^{\boldsymbol{j_0}}_{\boldsymbol{i_0,j_0}} = \left(\sum_{s=1}^{N_s-1} (N_s - s) \left(\boldsymbol{Q}^{\boldsymbol{j_0}}_{\boldsymbol{i_0,j_0}} \right)^{s-1} \boldsymbol{D}^{\boldsymbol{j_0}}_{\boldsymbol{i_0,j_0}} \right)^T \boldsymbol{h}_{\boldsymbol{i_0,j_0}} + \left(\widetilde{\boldsymbol{D}}^{\boldsymbol{j_0}}_{\boldsymbol{i,j_0}} \right)^{\boldsymbol{T}} \boldsymbol{e}^{\boldsymbol{j_0}}_{\boldsymbol{i-1,j}} \, \delta_{j_0,A} \tag{5}$$

where $\boldsymbol{h}_{\boldsymbol{i,j_0}} = (h_{i,j_0}, 0, \ldots, 0)^T$. Finally, for $j_0 = \{1, \ldots, A\}$:

$$\boldsymbol{e}^{\boldsymbol{j_0}}_{\boldsymbol{1,j_0}} = \left(\sum_{s=0}^{N_s-1} \left(\boldsymbol{Q}^{\boldsymbol{j_0}}_{\boldsymbol{i,j_0}} \right)^s \right)^T \boldsymbol{h}_{\boldsymbol{1,j_0}} \tag{6}$$

where $\boldsymbol{h}_{\boldsymbol{1,j_0}} = (h_{1,j_0}, 0, \ldots, 0)^T$.

The above equations can be understood starting considering the infection intervals $(t_{i-1}, t_i]$ and $(a_{j_0-1}, a_{j_0}]$; h_{i,j_0} new infections occur at the beginning of each of the N_s constituent sub-intervals.

Newly infected individuals can either remain undiagnosed throughout or be diagnosed in one of the successive sub-intervals $(t_{i-1,s}, t_{i,s}]$ and $(a_{j_0-1,s}, a_{j_0,s}]$, $s = \{2, \ldots, N_s\}$. The probability to remaining undiagnosed is $(\boldsymbol{Q}^{\boldsymbol{j_0}}_{\boldsymbol{i,j_0}})^{\boldsymbol{N_s-1}}$. Diagnosis may occur in the second sub-interval ($s = 2$) with probability $\boldsymbol{D}^{\boldsymbol{j_0}}_{\boldsymbol{i,j_0}}$, in the third sub-interval ($s = 3$, probability $\boldsymbol{Q}^{\boldsymbol{j_0}}_{\boldsymbol{i,j_0}} \boldsymbol{D}^{\boldsymbol{j_0}}_{\boldsymbol{i,j_0}}$) and so on, up to the $s = N_s$ sub-interval, with probability $(\boldsymbol{Q}^{\boldsymbol{j_0}}_{\boldsymbol{i,j_0}})^{N_s-2} \boldsymbol{D}^{\boldsymbol{j_0}}_{\boldsymbol{i,j_0}}$. Similarly infections in the second sub-interval can remain undiagnosed throughout $(t_{i-1,s}, t_{i,s}]$ and $(a_{j_0-1,s}, a_{j_0,s}]$, $s = \{3, \ldots, N_s\}$ with probability $(\boldsymbol{Q}^{\boldsymbol{j_0}}_{\boldsymbol{i,j_0}})^{N_s-2}$. Alternatively, diagnoses can occur in the $s = 3$ subinterval (probability $\boldsymbol{D}^{\boldsymbol{j_0}}_{\boldsymbol{i,j_0}}$) and so on, up to the $s = N_s$ subinterval (probability $(\boldsymbol{Q}^{\boldsymbol{j_0}}_{\boldsymbol{i,j_0}})^{N_s-3} \boldsymbol{D}^{\boldsymbol{j_0}}_{\boldsymbol{i,j_0}}$). This is generalized in Equations 4 and 5.

After $(t_{i-1}, t_i]$ and $(a_{j_0-1}, a_{j_0}]$ no further infections occur. Equations 1 and 2 describe the expected number of individuals from the infected cohort in the model states over the successive time and age intervals. For instance, consider an individual remaining undiagnosed throughout the successive "coarse" intervals (*i.e.* $(t_i, t_{i+1}]$ and $(a_{j_0}, a_{j_0+1}]$). To do so he must remain undiagnosed in each of the N_s constituent sub-intervals. This equates to raising the transition matrix $\boldsymbol{Q}^{\boldsymbol{j_0}}_{\boldsymbol{i+1,j_0+1}}$ to the power of N_s. Instead, to be diagnosed within the "same" coarse intervals, the diagnosis must have occurred in one of the N_s sub-intervals: either in in the first sub-interval (probability $\boldsymbol{D}^{\boldsymbol{j_0}}_{\boldsymbol{i+1,j_0+1}}$), or in the second sub-interval (probability $\boldsymbol{Q}^{\boldsymbol{j_0}}_{\boldsymbol{i+1,j_0+1}} \boldsymbol{D}^{\boldsymbol{j_0}}_{\boldsymbol{i+1,j_0+1}}$) etcetera.

1.3 Back-calculation on different age and time scales

Here we relax the assumption that time and age are measured on the same scale, which has been central so far, as surveillance data are often available on a larger age scale than time scale. Data could be aggregated to have equal scales, but this would entail a loss of information.

Intervals $(t_{i-1}, t_i]$, $i = \{1, \dots T\}$, and $(a_{j-1}, a_j], j = \{1, \dots, A\}$, are now defined so that the length of N_a time-intervals is equal to the length of one age-interval (*e.g.* $N_a = 4$ for a quarterly time scale and a yearly age scale). Infected individuals become one age-interval older in the beginning of the $(N_a + 1)^{\text{th}}$, $(2N_a + 1)^{\text{th}}$, $\dots$ intervals.

To begin, assume that at most one transition between the model states is allowed per time interval. The dynamical Equations can be re-written as follows, for $i = \{2, \dots, T\}$, $j_0 = \{1, \dots, A-1\}$ and $j = \{j_0, \dots, min(A, j_0 + \left\lfloor \frac{i-\epsilon}{N_a} \right\rfloor)\}$, where ϵ is an infinitely small positive value:

$$\boldsymbol{e}^{\boldsymbol{j_0}}_{\boldsymbol{i,j}} = \begin{cases} \left(\boldsymbol{Q}^{\boldsymbol{j_0}}_{\boldsymbol{i,j}}\right)^T \boldsymbol{e}^{\boldsymbol{j_0}}_{\boldsymbol{i-1,j}} & \text{if } i \ \% \ N_a \neq 1 \\ \left(\boldsymbol{Q}^{\boldsymbol{j_0}}_{\boldsymbol{i,j}}\right)^T \boldsymbol{e}^{\boldsymbol{j_0}}_{\boldsymbol{i-1,j-1}} + \left(\boldsymbol{Q}^{\boldsymbol{j_0}}_{\boldsymbol{i,j}}\right)^T \boldsymbol{e}^{\boldsymbol{j_0}}_{\boldsymbol{i-1,j}} \delta_{j,A} & \text{if } i \ \% \ N_a = 1 \end{cases} \tag{7}$$

$$\boldsymbol{\mu}^{\boldsymbol{j_0}}_{\boldsymbol{i,j}} = \begin{cases} \left(\boldsymbol{D}^{\boldsymbol{j_0}}_{\boldsymbol{i,j}}\right)^T \boldsymbol{e}^{\boldsymbol{j_0}}_{\boldsymbol{i-1,j}} & \text{if } i \ \% \ N_a \neq 1 \\ \left(\boldsymbol{D}^{\boldsymbol{j_0}}_{\boldsymbol{i,j}}\right)^T \boldsymbol{e}^{\boldsymbol{j_0}}_{\boldsymbol{i-1,j-1}} + \left(\boldsymbol{D}^{\boldsymbol{j_0}}_{\boldsymbol{i,j}}\right)^T \boldsymbol{e}^{\boldsymbol{j_0}}_{\boldsymbol{i-1,j}} & \text{if } i \ \% \ N_a = 1 \end{cases} \tag{8}$$

where $i \ \% \ N_a = 1$ denotes the case where the remainder of the integer division of i by N_a is equal to one, identifying time intervals $i = \{N_a + 1, 2N_a + 1, \dots, \lfloor T/N_a \rfloor + 1\}$, at the beginning of which individuals age. $\boldsymbol{e}^{\boldsymbol{j_0}}_{\boldsymbol{i_0,j_0}}$ and $\boldsymbol{\mu}^{\boldsymbol{j_0}}_{\boldsymbol{i_0,j_0}}$, for $j = j_0$ are defined as in Equations (6) and (7) of the main paper and so $\boldsymbol{e_{1,j_0}}$, $j_0 = \{1, \dots A\}$.

The assumption of at most one move per time interval can be further relaxed by following Section 1.2, and replacing the matrices $\boldsymbol{Q}^{\boldsymbol{j_0}}_{\boldsymbol{i,j}}$ and $\boldsymbol{D}^{\boldsymbol{j_0}}_{\boldsymbol{i,j}}$ with $\widetilde{\boldsymbol{Q}}^{\boldsymbol{j_0}}_{\boldsymbol{i,j}}$ and $\widetilde{\boldsymbol{D}}^{\boldsymbol{j_0}}_{\boldsymbol{i,j}}$ (defined in Equation 3). $\boldsymbol{e}^{\boldsymbol{j_0}}_{\boldsymbol{i_0,j_0}}$ and $\boldsymbol{\mu}^{\boldsymbol{j_0}}_{\boldsymbol{i_0,j_0}}$ would be as in Equations 4 and 5.

Scenarios with a time scale wider than the age scale could also be considered by reversing the time and age indices used in this Section.

2 Splines

In Section 4.1 of the main paper we mention a number of splines, without however giving full mathematical details. These are described in the following sections.

2.1 Univariate Splines

A univariate spline is a flexible function $g(\boldsymbol{x}) : \mathbb{R} \rightarrow \mathbb{R}$ used for smoothly modelling the $\boldsymbol{x}$ - $\boldsymbol{y}$ relationship, where $\boldsymbol{y} = (y_1, \ldots, y_n)^T$, is a vector of n observations from the exponential family, with associated univariate $n \times 1$ vector of covariates $\boldsymbol{x} = (x_1, \ldots, x_n)^T$. Finally let $\boldsymbol{g} = (g(x_1), \ldots, g(x_n))^T$ be the $n \times 1$ vector of the spline's fitted values.

2.1.1 Optimal natural cubic splines

? first considered the following minimization problem, in the space of two times continuoulsy differentiable functions:

$$min \sum_{i=1}^{n} (\ y_i - g(x_i)\ )^2 + \lambda \int_{\mathbb{R}} \{g''(x)\}^2 dx \tag{9}$$

This objective function is a special case of the penalised likelihood criteria (Equation (14) in the main paper), where observations are assumed to be normally distributed. It represent a compromise between goodness of fit, as measured by the residual sum of squares, and the roughness of the curve, as measured by the integrated second derivative squared of $g(x)$. Again, λ is the smoothing parameter.

Theorem 1 *Equation 9 is uniquely minimized, in the space of continuously differentiable function in $\mathbb{R}$, by a natural cubic spline (NCS) with a knot for every unique x_i.*

? show that a NCS can be expressed as follows:

Definition 1 Consider a function $g(x) : [a, b] \rightarrow \mathbb{R}$, defined by a set of ordered knots $\boldsymbol{\kappa} = \{a = \kappa_1 < \kappa_2 < \cdots < \kappa_k = b\}$ and parameters $\alpha_1, \alpha_2, \delta_1, \ldots \delta_k$:

$$g(x) = \alpha_0 + \alpha_1 x + \frac{1}{12} \sum_{j=1}^{k} \delta_j |x - \kappa_j|^3$$

This is a NCS if the following constraints are satisfied:

$$\sum_{j=1}^{k} \delta_j = \sum_{j=1}^{k} \delta_j \kappa_j = 0$$

Note that the basis for a NCS is: $\left\{1, x, \frac{1}{12}|x - \kappa_1|^3, \ldots, \frac{1}{12}|x - \kappa_k|^3\right\}$.

The optimal NCS can be formulated within the penalised regression framework discussed in Section 4.1 of the main paper, by defining parameter vectors

$\boldsymbol{\alpha} = (\alpha_0, \alpha_1)^T$ and $\boldsymbol{\delta} = (\delta_1, \ldots, \delta_n)^T$, and matrices $\boldsymbol{T}$ and $\boldsymbol{E}$ respectively of dimension $n \times 2$ and $n \times n$:

$$\boldsymbol{T} = \begin{bmatrix} 1 & x_1 \\ \vdots & \vdots \\ 1 & x_n \end{bmatrix} \quad \boldsymbol{E} = \begin{bmatrix} \frac{1}{12}|x_1 - x_1|^3 & \cdots & \frac{1}{12}|x_1 - x_n|^3 \\ \vdots & \cdots & \vdots \\ \frac{1}{12}|x_n - x_1|^3 & \cdots & \frac{1}{12}|x_n - x_n|^3 \end{bmatrix} \tag{10}$$

so that the optimal NCS, with one knot per observation, can be written as follows:

$$\boldsymbol{g} = \boldsymbol{T\alpha} + \boldsymbol{E\delta} \quad \text{s.t} \quad \boldsymbol{T^T\delta} = \boldsymbol{0} \tag{11}$$

? (page 141), demonstrate that the roughness integral can be expressed in quadratic form:

$$\int_a^b \{g''(x)\}^2 dx = \boldsymbol{\delta^T E \delta}$$

Note that $\boldsymbol{T^T\delta} = \boldsymbol{0}$ imposes two constraints on $\boldsymbol{\delta}$, thus it would be simpler working with the vector $\boldsymbol{\delta'}$ having $n-2$ free parameters. $\boldsymbol{\delta}$ and $\boldsymbol{\delta'}$ can be linked via an orthogonal dimension (or rank) reduction matrix $\boldsymbol{Z}$, of size $n \times n-2$, so that $\boldsymbol{\delta} = \boldsymbol{Z\delta'}$ and the constraint term becomes $\boldsymbol{T^T Z\delta'} = \boldsymbol{T^T\delta} = 0$. By constraining $\boldsymbol{T^T Z}$ to be $\boldsymbol{0}$, the constraint is satisfied for any value $\boldsymbol{\delta'}$, which then becomes an unconstrained vector. The matrix $\boldsymbol{Z}$ is obtained via a QR decomposition (of $\boldsymbol{T}$), for further details see Section 4 of **?** and also **?**.

Then optimal NCS can be described in the penalised likelihood framework described in the paper (Equation (14)), where a spline is characterised by parameters $\boldsymbol{\beta}$ and matrices $\boldsymbol{X}$ and $\boldsymbol{S}$:

$$\boldsymbol{\beta}_{[n\times 1]} = \begin{bmatrix} \alpha_0 & \alpha_1 & \delta'_1 & \cdots & \delta'_{n-2} \end{bmatrix}^T \tag{12}$$

$$\boldsymbol{X}_{[n\times n]} = \left[\boldsymbol{T}_{[\boldsymbol{n}\times\boldsymbol{2}]} \middle| \, \boldsymbol{E}_{[\boldsymbol{n}\times\boldsymbol{n}]}\boldsymbol{Z}_{[\boldsymbol{n}\times\boldsymbol{n-2}]}\right] \tag{13}$$

$$\boldsymbol{S}_{[n\times n]} = \left[\begin{array}{c|c} \boldsymbol{0}_{[\boldsymbol{2}\times\boldsymbol{2}]} & \boldsymbol{0}_{[\boldsymbol{2}\times\boldsymbol{n-2}]} \\ \hline \boldsymbol{0}_{[\boldsymbol{n-2}\times\boldsymbol{2}]} & \boldsymbol{Z^T E Z}_{[\boldsymbol{n-2}\times\boldsymbol{n-2}]} \end{array}\right] \tag{14}$$

Optimal NCS require a knot, and therefore a parameter, per observation. These may be computationally challenging (in terms of both running time and numerical stability) to fit, especially when many observations are available. Thus *"low-rank"* splines, with fewer parameters than observations, are considered. Despite not having the optimality property of optimal NCS, these work well in practice and are described in the following Section 2.1.2 to 2.1.4.

2.1.2 Thin plate regression splines

? introduces thin plate regression splines which are an "optimal" low-rank approximation of optimal (or full-rank) NCS. The main idea is to introduce a dimension (or rank) reduction matrix $\boldsymbol{\Gamma_p}$ of size $n \times p$, relating the p parameters of a low-rank NCS to the n parameters of a full-rank NCS:

$$\boldsymbol{\delta} = \boldsymbol{\Gamma_p \delta_p} \tag{15}$$

Using the same data, the full and low rank splines yield different fitted values and penalise roughness differently. Such differences can be quantified and minimized by finding an "optimal" (in the sense defined by **?**) $\boldsymbol{\Gamma_p}$, which is shown to be equal to $\boldsymbol{U_p}$, obtained from the eigen-decomposition of matrix $\boldsymbol{E}$ (defined in Equation 10). Explicitly $\boldsymbol{E}$ (of size $n \times n$) can be expressed as the matrix product $\boldsymbol{UDU^T}$. $\boldsymbol{D}$ is a diagonal matrix, of dimension $n \times n$, with the absolute values of the eigenvalues of $\boldsymbol{E}$ sorted in ascending order along the main diagonal. $\boldsymbol{U}$ is the $n \times n$ matrix of eigenvectors. Let $\boldsymbol{U_p}$ be the $n \times p$ matrix consisting of the first p columns of $\boldsymbol{U}$ and $\boldsymbol{D_p}$ be the top left $p \times p$ submatrix of $\boldsymbol{D}$. $\boldsymbol{U_p}$ is a matrix of rank p, the columns of which form a p-dimensional orthonormal basis. Thus $\boldsymbol{U_p^T U_p = I_k}$, but $\boldsymbol{U_p U_p^T} \neq \boldsymbol{I}_n$, where $\boldsymbol{I}_l$ denotes an identity matrix of size l. Now:

$$\boldsymbol{g} = \boldsymbol{EU_p\delta_p - T\alpha} \quad \text{s.t} \quad \boldsymbol{T^T \Gamma_p \delta_p} = 0 \tag{16}$$

$$\boldsymbol{S} = \boldsymbol{U_p^T E U_p} \tag{17}$$

where $\boldsymbol{T}$ was defined in Equation 10. As discussed in the previous Section, the constrained minimisation problem can be turned into an unrestricted one by finding (via the QR decomposition of $\boldsymbol{T}$) an orthogonal matrix $\boldsymbol{Z}$ (size $p \times p-2$) so that $\boldsymbol{T^T U_p Z} = 0$ and $\boldsymbol{\delta_p = Z\delta'}$. Now thin plate regression splines can be expressed within the usual penalised regression framework (Equation (14) of the main paper), defining:

$$\boldsymbol{\beta}_{[p\times 1]} = \begin{bmatrix}\alpha_1 \; \alpha_2 \; \delta'_1 \; \cdots \; \delta'_{p-2}\end{bmatrix}^T \tag{18}$$

$$\boldsymbol{X}_{[n\times p]} = \begin{bmatrix}\boldsymbol{T}_{[n\times 2]} | \; \boldsymbol{E_{[n\times n]} U_{p[n\times p]} Z_{[p\times p-2]}}\end{bmatrix} \tag{19}$$

$$\boldsymbol{S}_{[p\times p]} = \left[\begin{array}{c|c} \boldsymbol{0_{[2\times 2]}} & \boldsymbol{0_{[2\times p-2]}} \\ \hline \boldsymbol{0_{[p-2\times 2]}} & \boldsymbol{Z^T D_k Z_{[p-2\times p-2]}} \end{array}\right] \tag{20}$$

Thin plate regression splines are defined in terms of the k largest eigenvectors, rather than a set of knots, avoiding having to explicitly choose the knots location. The number of parameters k shall be chosen to be adequately large to ensure sufficient flexibility, but not excessively large, to avoid computational waste.

2.1.3 Thin plate regression splines with linear shrinkage

All splines considered so far (optimal NCS, and thin plate regression splines) are made of a linear term $\boldsymbol{T\alpha}$ (*i.e.* $\alpha_0 + \alpha_1 x$) and a non-parametric term (*i.e.* $\boldsymbol{E\delta}$, often subject to reparameterisation).

As λ increases, the penalty matrix $\boldsymbol{S}$ shrinks the parameters $\boldsymbol{\delta}$ towards zero while the parameters $\boldsymbol{\alpha}$ are not subject to any penalty term (see Equations 14 and 20. Thus greater λ values shrink the spline towards a straight line (*i.e.* the unpenalised term) but not towards zero. **?**, within a context of variable selection, propose a strategy to penalise the null-space (*i.e.* the space of unpenalised coefficients $\boldsymbol{\alpha}$) allowing shrinkage to zero.

The eigendecomposition $\boldsymbol{S} = \boldsymbol{UDU^T}$ is considered, where $\boldsymbol{U}$ and $\boldsymbol{D}$ are as defined in Section 2.1.2. As there are two unpenalised coefficients (*i.e.* α_1, α_2), the last two eigenvalues are equal to zero. These are replaced by a small portion ϵ of the minimum strictly positive eigenvalue of $\boldsymbol{D}$, creating matrix $\boldsymbol{D'}$. The original penalty matrix $\boldsymbol{S}$ can be then replaced by $\boldsymbol{S'} = \boldsymbol{UD'U^T}$. This gives $\boldsymbol{S'} \approx \boldsymbol{S}$, so that the null-space is penalised.

? show that $\epsilon = 1/10$ works well in practice, thus the penalty imposed on the null space is smaller than the one imposed on the originally penalised space. Hence as λ increases the spline is first penalised towards a straight line, and then, if needed, the straight line is further shrunk to zero.

Thin plate regression splines with shrinkage can be expressed in the usual penalised likelihood framework. $\boldsymbol{\beta}$ and $\boldsymbol{X}$ are as defined in Equation 18 and 19, but $\boldsymbol{S}$ (Equation 20) is replaced by $\boldsymbol{S'}$, as defined above.

These splines are used as marginal splines for the *ptenstprs* spline discussed in the paper.

2.1.4 B and P-splines

?, building on the work by **?**, suggest a pragmatic alternative to construct a low-rank spline that is not based on approximations of optimal NCS (as in Section 2.1.2 and 2.1.3). They consider P-splines, which are B-splines (**?**) estimated within a penalised likelihood framework.

B-splines are low-rank polynomial splines characterised by a local basis, defined by degree d and $\boldsymbol{\kappa} = \{a = \kappa_1 < \cdots < \kappa_k = b\}$ (a,b $\in \mathbb{R}$) equally spaced internal knots dividing the domain $[a, b]$ into $k-1$ disjoint intervals. The basis function $B_i^d(x)$ is recursively defined:

$$B_i^d(x) = \frac{x - \kappa_{i-d}}{\kappa_i - \kappa_{i-d}} B_{i-1}^{d-1}(x) + \frac{\kappa_{i+1} - x}{\kappa_{i+1} - \kappa_{i+1-d}} B_i^{d-1}(x) \tag{21}$$

$$B_i^0(x) = \begin{cases} 1 & \text{for } \kappa_i \le x \le \kappa_{i+1} \\ 0 & \text{otherwise} \end{cases}$$

This recursion requires $2d$ extra knots outside of $[a, b]$ to be valid, yielding a total number of knots of $k + 2d$ and $k - 1 + d$ ($\equiv p$) B-spline bases $\left\{B_1^d(x), \ldots, B_{k-1+d}^d(x)\right\}$. A B-spline is a linear combination of these bases, so that:

- It is made of $d + 1$ polynomials of degree d.
- The polynomials join at the k inner knots.
- The derivative up to order $d - 1$ are continuous.
- $k - 1 + d$ polynomial bases are defined by the recursion.
- For every $x \in (a, b)$ only $d + 1$ bases are non-zero.
- For all x $\in [a, b]$: $\sum_{i=1}^{k-1+d} B_i^d(x) = 1$.

? define P-splines by estimating the coefficients of a B-spline subject to a penalty. If successive coefficients take similar values, the fitted spline is smooth. Consequently a difference matrix $\boldsymbol{D_r}$ of degree r is used as penalty matrix, where $r = 1$ and $r = 2$ are most commonly used:

$$\boldsymbol{D_1} = \begin{bmatrix} -1 & 1 & 0 & \cdots & 0 \\ 0 & -1 & 1 & \cdots & 0 \\ \vdots & \ddots & \ddots & \ddots & \vdots \\ 0 & \cdots & 0 & -1 & 1 \end{bmatrix} \qquad \boldsymbol{D_2} = \begin{bmatrix} -1 & 2 & -1 & 0 & \cdots & 0 \\ 0 & -1 & 2 & -1 & \cdots & 0 \\ \vdots & \ddots & \ddots & \ddots & \ddots & \vdots \\ 0 & \cdots & 0 & -1 & 2 & -1 \end{bmatrix}$$

The penalty matrix $\boldsymbol{S}$ is obtained by multiplying: $\boldsymbol{D_r^T D_r}$. For first and second order difference matrices the penalty term is equivalent to: $\boldsymbol{\beta^T D_1^T D_1 \beta} = \sum_{i=1}^{k-2+d}(\beta_{i+1} - \beta_i)^2$ and $\boldsymbol{\beta^T D_2^T D_2 \beta} = \sum_{i=1}^{k-3+d}(\beta_{i+2} - 2\beta_{i+1} + \beta_i)^2$.

A first order penalty shrinks the coefficients (and thus the spline) towards a common constant, as only two equal successive coefficients are not penalised. In contrast, a second order penalty shrinks coefficients towards a linear trend, as only three neighbouring coefficients forming a linear trend are unpenalised.

As before, P-splines of degree d, with a penalty matrix of order r, can be expressed within the usual penalised likelihood framework, where:

$$\boldsymbol{\beta}_{[p\times 1]} = \begin{bmatrix} \beta_1 & \cdots & \beta_p \end{bmatrix}^T \tag{22}$$

$$\boldsymbol{X}_{\boldsymbol{d}[\boldsymbol{n}\times\boldsymbol{p}]} = \begin{bmatrix} B_1^d(x_1) & \cdots & B_p^d(x_1) \\ \vdots & \vdots & \vdots \\ B_1^d(x_n) & \cdots & B_p^d(x_n) \end{bmatrix} \tag{23}$$

$$\boldsymbol{S}_{\boldsymbol{r}\,[p\times p]} = \left[\begin{array}{c|c} \mathbf{0}_{[r\times r]} & \mathbf{0}_{[r\times(p-r)]} \\ \hline \mathbf{0}_{[(p-r)\times r]} & (\boldsymbol{D_r^T D_r})_{[(p-r)\times(p-r)]} \end{array}\right] \tag{24}$$

d and r are suppressed for notational simplicity, so that $\boldsymbol{X} \equiv \boldsymbol{X_d}$ and $\boldsymbol{S} \equiv \boldsymbol{S_r}$. In the paper we consider $d = 3$ and $r = 1$, for the marginal splines of the *ptensbs* spline. **?** also considers the case for $d = 3$ and $r = 2$.

2.2 Bivariate splines

A bivariate spline is a flexible function $g(\boldsymbol{x}) : \mathbb{R}^2 \to \mathbb{R}$ used for smoothly modelling the $\boldsymbol{x}$ - $\boldsymbol{y}$ relationship, where $\boldsymbol{y} = (y_1, \ldots, y_n)^T$, is a vector of n observations from the exponential family, with associated covariates $\boldsymbol{x} = \{\boldsymbol{x}_1, \ldots, \boldsymbol{x}_n\}$, where $\boldsymbol{x}_i = (x_{1i}, x_{2i})^T$. $\boldsymbol{g} = (g(\boldsymbol{x}_1), \ldots, g(\boldsymbol{x}_n))^T$ denotes the $n \times 1$ vector of the spline's fitted values.

2.2.1 Optimal thin plate splines

? extends the univariate smoothing problem, posed in Section 2.1.1 to bivariate settings, by measuring roughness in $\mathbb{R}^2$ (*i.e.* in two dimensions) using the Laplacian quadratic integral. Hence the problem of finding a function $g(\boldsymbol{x})$, in the space of twice continuously differentiable functions, minimising the following criterion is considered:

$$min \sum_{i=1}^{n} (\ y_i - g(\boldsymbol{x}_i)\ )^2 + \lambda \int\int_{\mathbb{R}^2} \left(\frac{\partial^2 g(\boldsymbol{x})}{\partial x_1^2}\right)^2 + 2\left(\frac{\partial^2 g(\boldsymbol{x})}{\partial x_1 \partial x_2}\right)^2 + \left(\frac{\partial^2 g(\boldsymbol{x})}{\partial x_2^2}\right)^2 dx_1 dx_2 \tag{25}$$

Similarly to the univariate objective function (Equation 9), the above criterion is a special case of penalised regression (see Equation (14) in the main paper). The following theorem provides the solution to the minimization problem:

Theorem 2 *In the space of continuously differentiable functions in $\mathbb{R}^2$, Equation 25 is uniquely minimized by a thin plate spline with a knot at every unique $\boldsymbol{x}_i$.*

Definition 2 A thin plate spline (TPS) is a function $g(\boldsymbol{x}) : [a, b] \times [c, d] \to \mathbb{R}$ defined on a set of knots $\boldsymbol{\kappa} = \{\boldsymbol{\kappa}_1, \cdots, \boldsymbol{\kappa}_k\}$, where $\boldsymbol{\kappa}_i = (\kappa_{i1}, \kappa_{i2})$, so that:

$$g(\boldsymbol{x}) = \alpha_0 + \alpha_1 x_1 + \alpha_2 x_2 + \sum_{i=1}^{k} \delta_i \nu(||\boldsymbol{x}_i - \boldsymbol{\kappa}_i||)$$

subject to the following constraints:

$$\sum_{i=1}^{k} \delta_i = \sum_{i=1}^{k} \delta_i \kappa_{i1} = \sum_{i=1}^{k} \delta_i \kappa_{i2} = 0$$

where $||\cdot||$ denotes the Euclidean distance and $\nu(r)$ is a $\mathbb{R} \rightarrow \mathbb{R}$ distance function:

$$\nu(r) = \begin{cases} \frac{1}{16\pi} r^2 log(r^2) & \text{if } r > 0 \\ 0 & \text{otherwise} \end{cases}$$

A TPS basis is thus: $\{1, x_1, x_2, \nu(||\boldsymbol{x} - \boldsymbol{\kappa}_1||), \ldots, \nu(||\boldsymbol{x} - \boldsymbol{\kappa}_k||)\}$.

Thin plate splines with a knot per observation (referred to as *"optimal TP"* from now on) are optimal as if roughness is measured by the Laplacian integral, there is no smoother spline with equal (or better) goodness of fit. Note that optimal TPS are simply optimal NCS (Section 2.1.1) extended to bivariate settings.

Optimal TPS can be formulated within the penalised likelihood framework (Equation (14) of the main paper), by defining the parameter vectors $\boldsymbol{\alpha} = [\alpha_0, \alpha_1, \alpha_2]^T$ and $\boldsymbol{\delta} = [\delta_1, \ldots, \delta_n]^T$, and matrices $\boldsymbol{T}$ and $\boldsymbol{E}$ (of dimension $n \times 3$ and $n \times n$ respectively):

$$\boldsymbol{T} = \begin{bmatrix} 1 & x_{11} & x_{12} \\ \vdots & \vdots & \vdots \\ 1 & x_{n1} & x_{n2} \end{bmatrix} \qquad \boldsymbol{E} = \begin{bmatrix} \nu(||\boldsymbol{x}_1 - \boldsymbol{x}_1||) & \cdots & \nu(||\boldsymbol{x}_1 - \boldsymbol{x}_n||) \\ \vdots & \ldots & \vdots \\ \nu(||\boldsymbol{x}_n - \boldsymbol{x}_1||) & \cdots & \nu(||\boldsymbol{x}_n - \boldsymbol{x}_n||) \end{bmatrix} \tag{26}$$

It can be shown that the roughness integral can be written in a quadratic form (**?**):

$$\int\int_{\mathbb{R}^2} \left(\frac{\partial^2 g(\boldsymbol{x})}{\partial x_1^2}\right)^2 + 2\left(\frac{\partial^2 g(\boldsymbol{x})}{\partial x_1 \partial x_2}\right)^2 + \left(\frac{\partial^2 g(\boldsymbol{x})}{\partial x_2^2}\right)^2 dx_1 dx_2 = \boldsymbol{\delta}^{\boldsymbol{T}} \boldsymbol{E} \boldsymbol{\delta} \tag{27}$$

Then parameters $\boldsymbol{\beta}$ and matrices $\boldsymbol{X}$ and $\boldsymbol{S}$ can be defined as in Equations 12 to 14 (Section 2.1.1), using the matrices $\boldsymbol{T}$ and $\boldsymbol{E}$ defined above.

As for NCS, TPS are computationally inefficient, and similar results can be obtained with low-rank splines having fewer parameters than knots.

2.2.2 Knots based thin plate splines

Optimal TPS are a special case of TPS, where a knot is placed at each observation; low-rank TPS can be defined based on a set of knots that is smaller than the set of observations. The number of knots chosen must be large enough, to ensure sufficient flexibility, but not excessively large, to avoid computational waste. Sensitivity analysis to the number of knots and their location is essential. Let $\boldsymbol{\kappa} = \{\boldsymbol{\kappa}_1 < \cdots < \boldsymbol{\kappa}_p\}$ be a set of knots ($p < n$), so that $\boldsymbol{\kappa}_i = (\kappa_{i1}, \kappa_{i2})$.

Let $\boldsymbol{\alpha} = [\alpha_0, \alpha_1, \alpha_2]^T$ and $\boldsymbol{\delta} = [\delta_1, \ldots, \delta_k]^T$ be parameter vectors and $\boldsymbol{T}$, $\boldsymbol{E}$ and $\boldsymbol{C}$ matrices of dimension $n \times 3$, $n \times p$ and $3 \times p$ respectively:

$$\boldsymbol{T} = \begin{bmatrix} 1 & x_{11} & x_{12} \\ \vdots & \vdots & \vdots \\ 1 & x_{n1} & x_{n2} \end{bmatrix} \tag{28}$$

$$\boldsymbol{E} = \begin{bmatrix} \nu(||\boldsymbol{x}_1 - \boldsymbol{\kappa}_1||) & \cdots & \nu(||\boldsymbol{x}_1 - \boldsymbol{\kappa}_p||) \\ \vdots & \ldots & \vdots \\ \nu(||\boldsymbol{x}_n - \boldsymbol{\kappa}_1||) & \cdots & \nu(||\boldsymbol{x}_n - \boldsymbol{\kappa}_p||) \end{bmatrix} \tag{29}$$

$$\boldsymbol{C} = \begin{bmatrix} 1 & 1 & \cdots & 1 \\ \kappa_{11} & \kappa_{12} & \cdots & \kappa_{1k} \\ \kappa_{21} & \kappa_{22} & \cdots & \kappa_{2k} \end{bmatrix} \tag{30}$$

Now:

$$\boldsymbol{g} = \boldsymbol{T}\boldsymbol{\alpha} + \boldsymbol{E}\boldsymbol{\delta} \quad \text{s.t} \quad \boldsymbol{C}\boldsymbol{\delta} = \boldsymbol{0}$$

Now consider the $p \times p - 3$ matrix $\boldsymbol{Z}$ obtained via a QR decomposition of $\boldsymbol{C}$, so that $\boldsymbol{\delta} = \boldsymbol{Z}\boldsymbol{\delta'}$ and $\boldsymbol{C}\boldsymbol{Z} = \boldsymbol{0}$. $\boldsymbol{Z}$ links the vector $\boldsymbol{\delta}$ of k coefficients (subject to three constraints) to the vector $\boldsymbol{\delta'}$ of $k-3$ unconstrained coefficients. Now the spline can be rewritten in the usual penalised likelihood framework, where:

$$\boldsymbol{\beta}_{[p\times 1]} = \left[\alpha_0 \ \alpha_1 \ \alpha_2 \ \delta'_1 \cdots \delta'_{p-3}\right]^T \tag{31}$$

$$\boldsymbol{X}_{[n\times p]} = \left[\boldsymbol{T}_{[\boldsymbol{n}\times\boldsymbol{3}]} | \ \boldsymbol{E}_{[\boldsymbol{n}\times\boldsymbol{k}]}\boldsymbol{Z}_{[\boldsymbol{p}\times\boldsymbol{p}-\boldsymbol{3}]}\right] \tag{32}$$

$$\boldsymbol{S}_{[p\times p]} = \left[\begin{array}{c|c} \boldsymbol{0}_{[\boldsymbol{3}\times\boldsymbol{3}]} & \boldsymbol{0}_{[\boldsymbol{3}\times\boldsymbol{p}-\boldsymbol{3}]} \\ \hline \boldsymbol{0}_{[\boldsymbol{p}-\boldsymbol{3}\times\boldsymbol{3}]} & \boldsymbol{Z}^T\boldsymbol{E}\boldsymbol{Z}_{[\boldsymbol{p}-\boldsymbol{3}\times\boldsymbol{p}-\boldsymbol{3}]} \end{array}\right] \tag{33}$$

These splines are denoted *tps* in the paper.

2.2.3 Thin plate regression splines

Thin plate regression splines (Section 2.1.2) can be further extended to bivariate settings, by defining the coefficients $\boldsymbol{\beta}$ (Equation 18), and the design $\boldsymbol{X}$ (Equation 19) and penalty $\boldsymbol{S}$ matrices (Equation 20). The only difference with respect to Section 2.1.2 is that we the matrices $\boldsymbol{E}$ and $\boldsymbol{T}$ given in Equation 26 to construct the aforementioned matrices.

2.2.4 Thin plate regression splines with shrinkage

Shrinkage of thin plate regression splines towards zero can be further achieved by imposing a penalty on the null-space (*i.e.* the $\boldsymbol{\alpha}$ coefficients). Instructions are given in Section 2.1.3, with the only difference that the $\boldsymbol{E}$ and $\boldsymbol{T}$ matrices employed, are defined in Equation 26. These splines are denoted *tprs* in the paper.

2.2.5 Tensor Product splines

Tensor product splines are here constructed following **?**, generalising the approach of **?**.

Tensor product splines are obtained by multiplying two univariate spline bases, each separately defined on the marginal dimensions (*i.e.* x_1 and x_2). This is a pragmatic approach for constructing bivariate splines; unlike bivariate TPS splines (Section 2.2.1 to 2.2.4), these are not motivated by the smoothing criterion in Equation 25 depending on the Laplacian quadratic integral, which assumes isotropy, *i.e.* roughness is equally penalised in the dimensions x_1 and x_2. Tensor product splines relax this assumption.

We start by considering two univariate splines $g_1(x_1) : [a, b] \to \mathbb{R}$ and $g_2(x_2) : [c, d] \to \mathbb{R}$, with bases $\{B_{11}(x_1), \cdots, B_{1p_1}(x_1)\}$ and $\{B_{21}(x_2), \cdots, B_{2p_2}(x_2)\}$, coefficients $\boldsymbol{\beta_1} = [\beta_{11}, \ldots, \beta_{1p_1}]^T$ and $\boldsymbol{\beta_2} = [\beta_{21}, \ldots, \beta_{2p_2}]^T$, design matrices $\boldsymbol{X_{(1)}}$ and $\boldsymbol{X_{(2)}}$ (of dimension $n \times p_1$ and $n \times p_2$ respectively), and penalty matrices $\boldsymbol{S_{(1)}}$ and $\boldsymbol{S_{(2)}}$ (of dimension $p_1 \times p_1$ and $p_2 \times p_2$ respectively). For $i = \{1, \ldots, p_1\}$ and $j = \{1, \ldots, p_2\}$ the tensor product basis is equal to:

$$B_{i,j}(x_1, x_2) = B_{1i}(x_1)B_{2j}(x_2)$$

Hence the tensor product design matrix $\boldsymbol{X}$, of dimension $n \times (p_1 p_2)$, is:

$$\boldsymbol{X} = \begin{bmatrix} B_{1,1}(x_{11}, x_{12}) & \cdots & B_{p_1,1}(x_{11}, x_{12}) & \cdots & B_{1,p_2}(x_{11}, x_{12}) & \cdots & B_{p_1,p_2}(x_{11}, x_{12}) \\ \vdots & \vdots & \vdots & \vdots & \vdots & \vdots & \vdots \\ B_{1,1}(x_{n1}, x_{n2}) & \cdots & B_{p_1,1}(x_{n1}, x_{n2}) & \cdots & B_{1,p_2}(x_{n1}, x_{n2}) & \cdots & B_{p_1,p_2}(x_{n1}, x_{n2}) \end{bmatrix}$$

The ith row of $\boldsymbol{X}$, denoted $\boldsymbol{X_{i\cdot}}$, can be alternatively obtained from the Kroenecker product of the ith rows of the marginal P-splines design matrices, $\boldsymbol{X}_{(1)i\cdot}$ and $\boldsymbol{X}_{(2)i\cdot}$ respectively, yielding a $(p_1 p_2) \times 1$ vector:

$$\boldsymbol{X_{i\cdot}} = \boldsymbol{X}_{(1)i\cdot} \otimes \boldsymbol{X}_{(2)i\cdot} \qquad (34)$$

The $(p_1 p_2) \times 1$ parameter vector of the tensor product spline is $\boldsymbol{\beta} = [\beta_{1,1}, \cdots, \beta_{p_1,1}, \cdots, \beta_{1,p_2}, \cdots, \beta_{p_1,p_2}]^T$.

The overall roughness of the tensor product spline can be quantified based on the idea that we know how to measure roughness marginally (via the specification of $\boldsymbol{S_{(1)}}$ and $\boldsymbol{S_{(2)}}$). For a fixed x_2, $J_1(g(x_1|x_2)) = \boldsymbol{\beta_1^T S_{(1)} \beta_1}$ quantifies roughness with respect to the x_1 dimension. Evaluating $J_1(g(x_1|x_2))$ for infinitely many fixed x_2 and taking its average over the x_2 points yields the overall roughness with respect to x_1. Roughness in the x_2 direction is similarly measured, fixing x_1 instead. The overall roughness of $g(x_1, x_2)$ is mathematically expressed as:

$$J(g(\boldsymbol{x})) = \lambda_{(1)} \int_c^d J_1\left(g_1(x_1|x_2)\right) dx_2 + \lambda_{(2)} \int_a^b J_{x_2}\left(g_2(x_2|x_1)\right) dx_1 \qquad (35)$$

This integral can not be analytically evaluated; a discrete approximation can be derived by reparameterising the spline in terms of the values of the functions on a regular grid (see **?** page 99 for a picture). A set of equidistant values, denoted as $\boldsymbol{x}_1^\star = \{x_{11}^\star, \ldots, x_{1p_1}^\star\}$ and $\boldsymbol{x}_2^\star = \{x_{21}^\star, \ldots, x_{2p_2}^\star\}$ respectively, is constructed in the x_1 and x_2 dimensions. It can be shown that (**?**):

$$\int_c^d J_1\left(g_1(x_1|x_2)\right) dx_2 \approx$$

$$h_1 \sum_{j=1}^{p_2} J_1\left(g_1(x_1|x_{2j}^\star)\right) = h_1 \left(\boldsymbol{\beta}^T \left(\boldsymbol{A_1^{-T}} \boldsymbol{S_{(1)}} \boldsymbol{A_1}\right) \otimes \boldsymbol{I_{p_2}} \boldsymbol{\beta}\right) \tag{36}$$

where $\boldsymbol{A_1}$ is a $p_1 \times p_1$ matrix with entries $(\boldsymbol{A_1})_{ij} = B_{1i}(x_{1j}^\star)$, and h_1 is a constant of proportionality to account for the spacing of $\boldsymbol{x_1^\star}$. The integral for $J_2\left(g_2(x_2|x_1)\right)$ can be similarly approximated, by defining instead h_2 and a $p_2 \times p_2$ matrix $\boldsymbol{A_2}$, with entries $(\boldsymbol{A_2})_{ij} = B_{2i}(x_{2j}^\star)$.

Tensor product splines can be expressed within the usual penalised regression framework where $\lambda_1 = h_1\lambda_{(1)}$, $\lambda_2 = h_2\lambda_{(2)}$ and:

$$\boldsymbol{\beta} = [\beta_{1,1}, \cdots, \beta_{p_1,1}, \cdots, \beta_{1,p_2}, \cdots, \beta_{p_1,p_2}]^T \tag{37}$$

$$\boldsymbol{S_1} = \left(\boldsymbol{A_1^{-T}} \boldsymbol{S_{(1)}} \boldsymbol{A_1}\right) \otimes \boldsymbol{I_{p_2}} \tag{38}$$

$$\boldsymbol{S_2} = \boldsymbol{I_{p_1}} \otimes \left(\boldsymbol{A_2^{-T}} \boldsymbol{S_{(2)}} \boldsymbol{A_2}\right) \tag{39}$$

In the paper we specifically consider univariate thin plate regression splines (see Section 2.2.4) and cubic B-splines with a first order difference penalty (see Section 2.1.4)

2.3 Reparameterisations for increased computational efficiency

Consider a spline characterised by parameters $\boldsymbol{\beta}$ (size p) and design $\boldsymbol{X}$ and penalty $\boldsymbol{S}$ matrices, respectively of size $n \times p$ and $p \times p$. Two reparameterisations can be employed to increase the computational efficiency of HMC sampling.

2.3.1 Centering reparameterisation

Any spline, as defined above, can be made subject to a sum to zero constraint, *i.e.* $\sum_{i=1}^n g(x_i) = 0$). In matrix notation this is equivalent to

$$\boldsymbol{1^T} \boldsymbol{X}\boldsymbol{\beta} = 0 \tag{40}$$

Where $\mathbf{1}$ is a $n \times 1$ vector. The above constraint can be integrated using the usual QR decomposition (of $\mathbf{1^T X}$) approach. An orthogonal matrix $\boldsymbol{Z}$, of dimension $p \times (p-1)$, is found so that $\boldsymbol{\beta} = \boldsymbol{Z\beta'}$ and $\mathbf{1^T}\boldsymbol{XZ} = 0$. After reparameterisation, the quadratic penalty matrix for $\boldsymbol{\beta'}$, of size $(p-1)\times(p-1)$, is $\boldsymbol{Z^T S Z}$.

Notice that in integrating such constraint results in loosing a degree of freedom. This is compensated by the introduction of a global intercept α. Thus the resulting reparametrized spline has p parameters $\tilde{\boldsymbol{\beta}} = \begin{bmatrix}\alpha\ \boldsymbol{\beta'}\end{bmatrix}$, design matrix $\tilde{\boldsymbol{X}} = \begin{bmatrix}\mathbf{1} |\ \boldsymbol{XZ}\end{bmatrix}$ of size $p \times p$ and finally $\tilde{\boldsymbol{S}} = \begin{bmatrix}\mathbf{0}^T \\ \boldsymbol{Z^T S Z}\end{bmatrix}$, where $\mathbf{0}$ is a vector of zeroes of size p.

Further details about this re-parameterisation are available in **?**, Section 4.2.

2.3.2 Identity precision reparameterisation

Splines with a single penalty matrix $\boldsymbol{S}$, *i.e.* all univariate splines and bivariate thin plate (regression) splines (with shrinkage), can be reparametrized to have an identity penalty matrix.

Recall that $\boldsymbol{S}$ is reinterpreted, within a Bayesian framework, as the precision matrix of a multivariate normal prior on coefficients $\boldsymbol{\beta}$. Hence having $\boldsymbol{S} = \boldsymbol{I}$ leads to i.i.d Normal priors for the $\boldsymbol{\beta}$ components. This reparameterisation leads to faster HMC updating in our experience.

Consider any spline where $\rho \leq p$ is the rank of $\boldsymbol{S}$. If some of the $\boldsymbol{\beta}$ coefficients are unpenalised, then $\rho < p$. Denote $\boldsymbol{\beta_U}$ and $\boldsymbol{\beta_P}$, of size $p-\rho$ and ρ, unpenalised and penalised coefficients.

Apply the eigendecomposition $\boldsymbol{S} = \boldsymbol{UDU^T}$. $\boldsymbol{D}$ is a diagonal matrix, with entries being the eigenvalues of $\boldsymbol{S}$ sorted in ascending order and $\boldsymbol{U}$ is the matrix of corresponding eigenvectors. Due to positive semi-definiteness of $\boldsymbol{S}$ all eigenvalues are positive, with the exception of $p-\rho$ zero eigenvalues. Further let $\boldsymbol{\Lambda}$ be a diagonal matrix, with entries $\Lambda_{ii} = \sqrt{D_{ii}}$ so that $\boldsymbol{D} = \boldsymbol{\Lambda^T \Lambda}$. The penalty becomes:

$$\lambda\boldsymbol{\beta^T S \beta} = \lambda\boldsymbol{\beta^T U D U^T \beta} = \lambda\boldsymbol{\beta^T U \Lambda^T \Lambda U^T \beta} \tag{41}$$

Where:

$$\boldsymbol{U\Lambda^T \Lambda U^T} = \left[\begin{array}{c|c} \boldsymbol{I}_{[\rho\times\rho]} & \mathbf{0}_{[\rho\times p-\rho]} \\ \hline \mathbf{0}_{[p-\rho\times p-\rho]} & \mathbf{0}_{[p-\rho\times p-\rho]} \end{array}\right]$$

Now let $\boldsymbol{\beta'} = \boldsymbol{\Lambda U^T \beta}$ and notice that the penalty term is equivalent to:

$$\boldsymbol{\beta^T U \Lambda^T \Lambda U^T \beta} = \boldsymbol{\beta_P^T I \beta_P}$$

By orthogonality of $\boldsymbol{U}$ it holds that:

$$\boldsymbol{\beta} = (\boldsymbol{\Lambda U^T})^{-1}\boldsymbol{\beta'} = (\boldsymbol{U^T})^{-1}(\boldsymbol{\Lambda})^{-1}\boldsymbol{\beta'} = (\boldsymbol{U}^{-1})^{-1}\boldsymbol{\Lambda}^{-1}\boldsymbol{\beta'} = \boldsymbol{U\Lambda}^{-1}\boldsymbol{\beta'}$$

Hence the original design and penalty matrices of the spline are now reparametrized as:

$$\boldsymbol{X\beta} = \boldsymbol{XU\Lambda^{-1}\beta'} \qquad \lambda\boldsymbol{\beta^T S\beta} = \lambda\boldsymbol{\beta_P^T I\beta_P}$$

Further details about this reparameterisation are available in **?**. An improper flat prior is assigned to unpenalised coefficients $\boldsymbol{\beta_U}$. For computational efficiency reasons, it has been suggested to replace the flat prior by a vague proper prior, usually Normal for $\beta'_{Uj} \sim N(0, 1/\lambda_0)$. Hence the original penalty is now replaced by the following approximation:

$$\lambda\boldsymbol{\beta^T S\beta} \approx \lambda\boldsymbol{\beta_P^T I\beta_P} + \lambda_0\boldsymbol{\beta_U^T I\beta_U} \tag{42}$$

3 Further information on the simulation study

3.1 A note on the scale used

To avoid excessively long running times, we have run the simulation study using a yearly time and age-scale. However, a yearly scale is too coarse to make the assumption (as in Section 3.2 of the paper) that at most one event (*i.e.* diagnosis or progression) occur per time interval. Section 1.2 demonstrates how a yearly time scale can be construct from a "small" quarterly time scale. Hence we used Equations (1) to (5) in the supplementary material for simulating the model dynamics. For the same reasons of computational feasibility we considered the epidemic from an intermediate (and not the starting point). The dynamics equation can be derived as discussed in Section 1.1

3.2 Values for the expected number of initially undiagnosed infections $\boldsymbol{\pi}$

Age at t_1	Stage1 ($CD4 \geq 500$)	Stage2 ($500 < CD4 \leq 350$)	Stage3 ($350 < CD4 \leq 200$)	Stage4 ($CD4 > 200$)
1	8.36	1.75	1.75	0.31
2	13.14	3.90	3.90	1.16
3	15.86	5.91	5.91	2.32
4	17.41	7.56	7.56	3.54
5	19.84	11.06	11.06	6.64
6	21.24	13.74	13.74	9.34
7	22.04	15.74	15.74	11.56
8	22.44	17.08	17.08	13.17
9	22.62	17.97	17.97	14.30
10	22.74	18.81	18.81	15.43

11	46.25	24.55	24.55	17.30
12	59.51	31.31	31.31	20.54
13	66.91	37.09	37.09	23.91
14	71.01	41.83	41.83	27.44
15	71.96	43.54	43.54	28.93
16	72.42	44.86	44.86	30.23
17	72.61	45.83	45.83	31.28
18	72.60	46.48	46.48	32.04
19	72.51	46.91	46.91	32.56
20	72.33	47.06	47.06	32.74
21	72.13	47.19	47.19	32.89
22	71.93	47.28	47.28	33.01
23	71.72	47.33	47.33	33.07
24	71.50	47.37	47.37	33.11
25	71.28	47.37	47.37	33.09
26	71.05	47.36	47.36	33.07
27	70.82	47.34	47.34	33.03
28	70.59	47.32	47.32	33.00
29	70.35	47.29	47.29	32.96
30	70.11	47.26	47.26	32.91
31	39.80	40.21	40.21	31.53
32	23.02	31.72	31.72	28.02
33	13.74	24.04	24.04	23.40
34	8.59	17.83	17.83	18.68
35	5.78	13.19	13.19	14.53
36	4.18	9.72	9.72	11.02
37	3.27	7.18	7.18	8.21
38	2.78	5.51	5.51	6.23
39	2.53	4.42	4.42	4.87
40	2.37	3.62	3.62	3.82
41	2.26	2.99	2.99	2.97
42	2.19	2.50	2.50	2.28
43	2.17	2.42	2.42	2.16
44	2.15	2.35	2.35	2.06
45	2.14	2.33	2.33	2.03
46	2.12	2.31	2.31	2.01
47	2.11	2.30	2.30	2.00
48	2.10	2.28	2.28	1.98
49	2.08	2.27	2.27	1.97
50	2.07	2.26	2.26	1.95
51	2.06	2.24	2.24	1.94
52	5.88	11.43	11.43	11.48

Table 1: Values chosen for the expected number of initially undiagnosed individuals $\boldsymbol{\pi}$, for each CD4 undiagnosed state and age at time 1.

3.3 Values the progression probabilities $\boldsymbol{Q}$

Age at inf a_0	Stage1 ($CD4 \geq 500$)	Stage2 ($500 < CD4 \leq 350$)	Stage3 ($350 < CD4 \leq 200$)	Stage4 ($CD4 > 200$)
1	0.09	0.12	0.11	0.14
2	0.09	0.12	0.11	0.14
3	0.09	0.12	0.11	0.14
4	0.09	0.12	0.11	0.14
5	0.09	0.12	0.11	0.14
6	0.09	0.12	0.11	0.14
7	0.09	0.12	0.11	0.14
8	0.09	0.12	0.11	0.14
9	0.09	0.12	0.11	0.14
10	0.09	0.12	0.11	0.14
11	0.09	0.12	0.11	0.14
12	0.09	0.12	0.11	0.14
13	0.09	0.12	0.11	0.15
14	0.09	0.12	0.11	0.15
15	0.09	0.12	0.12	0.15
16	0.09	0.12	0.12	0.15
17	0.09	0.12	0.12	0.15
18	0.09	0.12	0.12	0.15
19	0.09	0.12	0.12	0.15
20	0.10	0.12	0.12	0.15
21	0.10	0.12	0.12	0.15
22	0.10	0.12	0.12	0.15
23	0.10	0.12	0.12	0.15
24	0.10	0.12	0.12	0.15
25	0.10	0.12	0.12	0.15
26	0.10	0.12	0.12	0.15
27	0.10	0.12	0.12	0.15
28	0.10	0.12	0.12	0.15
29	0.10	0.12	0.12	0.16
30	0.10	0.12	0.12	0.16
31	0.10	0.12	0.12	0.16
32	0.10	0.12	0.12	0.16
33	0.10	0.12	0.12	0.16
34	0.10	0.12	0.13	0.16
35	0.10	0.12	0.13	0.16
36	0.10	0.12	0.13	0.16
37	0.10	0.12	0.13	0.16
38	0.10	0.12	0.13	0.16
39	0.10	0.12	0.13	0.17

40	0.10	0.12	0.13	0.17
41	0.10	0.12	0.13	0.17
42	0.10	0.13	0.13	0.17
43	0.10	0.13	0.14	0.17
44	0.10	0.13	0.14	0.17
45	0.10	0.13	0.14	0.18
46	0.11	0.13	0.14	0.18
47	0.11	0.13	0.14	0.18
48	0.11	0.13	0.14	0.18
49	0.11	0.13	0.14	0.18
50	0.11	0.13	0.15	0.19
51	0.11	0.13	0.15	0.19
52	0.11	0.13	0.15	0.19

Table 2: Values chosen for the progression probabilities $\boldsymbol{\mathcal{Q}}$, for each CD4 undiagnosed state and age at time 1

3.4 Values of the diagnosis probabilities $\boldsymbol{\mathcal{D}}$

Time a_0	Stage1 ($CD4 \geq 500$)	Stage2 ($500 < CD4 \leq 350$)	Stage3 ($350 < CD4 \leq 200$)	Stage4 ($CD4 > 200$)
1	0.04	0.04	0.05	0.07
2	0.04	0.04	0.06	0.09
3	0.04	0.05	0.06	0.10
4	0.03	0.04	0.07	0.11
5	0.03	0.04	0.06	0.13
6	0.04	0.04	0.07	0.15
7	0.04	0.04	0.07	0.15
8	0.04	0.04	0.08	0.17
9	0.04	0.05	0.08	0.17
10	0.04	0.05	0.08	0.18
11	0.04	0.06	0.09	0.19
12	0.05	0.05	0.09	0.19
13	0.06	0.06	0.09	0.20
14	0.06	0.06	0.10	0.22
15	0.05	0.06	0.10	0.23
16	0.06	0.07	0.11	0.22
17	0.06	0.08	0.10	0.24
18	0.07	0.09	0.11	0.25
19	0.08	0.09	0.11	0.26
20	0.08	0.08	0.12	0.28

Table 3: Values chosen for the diagnosis probabilities $\boldsymbol{\mathcal{D}}$, for each CD4 undiagnosed state and age at time 1

3.5 Priors employed

Recall that the log-infection process $\boldsymbol{\gamma}$ is modelled using a bivariate spline (among those discussed in Table 1 of the paper). $\boldsymbol{\gamma} = \boldsymbol{X}\boldsymbol{\theta}$, with $\boldsymbol{X}$ representing the design matrix corresponding to the chosen type of spline. After the centering reparameterisations discussed in Section 2.3.1, we use the following prior to characterise the tensor product spline:

$$\begin{aligned} \theta_1 &\sim N(0, 30) \\ \boldsymbol{\theta}_{p-1} &\sim N_{p-1}\left(\mathbf{0}, (\lambda_1 \boldsymbol{S_1} + \lambda_2 \boldsymbol{S_2})^{-1}\right) \\ \sigma_1, \sigma_2 &\sim t_+(2, 200) \end{aligned} \tag{43}$$

where N_n denotes a nth dimensional multivariate Normal distribution, and $t_+(d, s)$ denotes a half-t distribution with d degrees of freedom and scale parameter s. Recall that θ_1 is a global intercept term and $\sigma_1 = \frac{1}{\lambda_1^2}$, $\sigma_2 = \frac{1}{\lambda_2^2}$ are smoothing parameters

Thin plate splines have a single penalty matrix $\boldsymbol{S}$ and are subject to both the centering and i.i.d Normal priors reparameterisations (Sections 2.3.1, 2.3.2) . They can hence be formulated as follows:

$$\begin{aligned} \theta_1 &\sim N(0, 30) \\ \theta_i &\sim N(0, \sigma^2), \quad i = \{2, \dots, p\} \\ \sigma &\sim t_+(2, 200) \end{aligned} \tag{44}$$

For both thin plate and tensor product splines: θ_1 is the global intercept, describing the mean number of log-expected infections per age and time interval. A very weakly informative prior is assigned to it, so that θ_1 lies with approximately 95% prior probability in the $[-60, 60]$ range. The choice of priors on the parameters $\boldsymbol{\theta}$ are dictated by the penalty term re-interpretation as a precision matrix. A diffuse half-t distribution with 2 degrees of freedom and scale parameter 200 is chosen as prior for the smoothing parameters, so that 95% of the prior density lies in the [0,400] region, reflecting a lack of knowledge for the $\boldsymbol{\theta}$ parameters (**?**). This, however, is a choice of prior to which outputs are particularly insensitive.

Prior distributions need to be further assigned to the diagnosis process $\boldsymbol{\mathcal{D}}(\boldsymbol{\delta})$:

$$\begin{aligned} \delta_{1,1} &\sim N(-3.2, 0.2), & \delta_{1,2} &\sim N(-3.2, 0.2), \\ \delta_{1,3} &\sim N(-3, 0.2), & \delta_{1,4} &\sim N(-2.5, 0.3) \end{aligned}$$

$$\sigma_k^2 \sim \Gamma(1,32), \quad k = \{1,2,3,4\}$$

The priors assigned to $\delta_{k,1}$ are weakly informative. The prior for σ_k^2 implies that the standard deviation lies with 95% prior probability in (0.03,0.335) and allows the logit-random walk to approximately vary by at most two standard deviations (*i.e.* 70%) between successive intervals.

4 The QR decomposition

This Appendix provides the details of the QR decomposition. For further details refer to **?**, page 46 and 334.

Let $\boldsymbol{X}$ be a $n \times m$ matrix, where $n \geq m$. $\boldsymbol{X}$ can always be decomposed as follows:

$$\boldsymbol{X} = \boldsymbol{Q} \begin{bmatrix} \boldsymbol{R} \\ \boldsymbol{0} \end{bmatrix} = \begin{bmatrix} \boldsymbol{Q_1} \ \boldsymbol{Q_2} \end{bmatrix} \begin{bmatrix} \boldsymbol{R} \\ \boldsymbol{0} \end{bmatrix} = \boldsymbol{Q_1 R_1} \tag{45}$$

Where $\boldsymbol{R}$ is an upper triangular $m \times m$ matrix, $\boldsymbol{0}$ is a $(n-m) \times m$ matrix and $\boldsymbol{Q}$ is a $n \times n$ orthogonal matrix. $\boldsymbol{Q}$ can be further split into $\boldsymbol{Q_1}$ (dimension $n \times m$) and $\boldsymbol{Q_2}$ (dimension $n \times n - m$), both with orthogonal columns.

Now consider the specific case where a linear (or spline) model $\boldsymbol{X\beta}$ is subject to the constraint $\boldsymbol{C\beta} = 0$. $\boldsymbol{C}$ is a $c \times m$ matrix, imposing c distinct constraints.

The aim is to to reparametrise the linear (or spline) model in terms of $\boldsymbol{\beta'}$ containing $m - c$ free parameters, rather than m parameters subject to c constraints. To do so it is necessary finding a matrix $\boldsymbol{Z}$, of dimension $m \times (m-c)$, so that:

$$\boldsymbol{\beta} = \boldsymbol{Z\beta'} \tag{46}$$

$$\boldsymbol{CZ} = 0 \tag{47}$$

After re-parameterisation in Equation 46, the constraint term becomes $\boldsymbol{CZ\beta'} = 0$. Then the condition in Equation 47 allows $\boldsymbol{\beta'}$ to take any value, while satisfying the constraint.

Finally, $\boldsymbol{Z}$ is constructed using the QR decomposition of $\boldsymbol{C^T}$.

$$\boldsymbol{C^T} = \begin{bmatrix} \boldsymbol{Q_1} \ \boldsymbol{Q_2} \end{bmatrix} \begin{bmatrix} \boldsymbol{R} \\ \boldsymbol{0} \end{bmatrix} \tag{48}$$

Now set $\boldsymbol{Z} = \boldsymbol{Q_2}$ and consider:

$$\boldsymbol{CZ} = \begin{bmatrix} \boldsymbol{R^T} \ \boldsymbol{0} \end{bmatrix} \begin{bmatrix} \boldsymbol{Q_1^T} \\ \boldsymbol{Z^T} \end{bmatrix} \boldsymbol{Z} = \begin{bmatrix} \boldsymbol{R^T} \ \boldsymbol{0} \end{bmatrix} \begin{bmatrix} \boldsymbol{0} \\ \boldsymbol{I} \end{bmatrix} = \boldsymbol{0} \tag{49}$$

$\boldsymbol{Q_1}$ and $\boldsymbol{Q_2}$ span different columns of the same orthogonal matrix, thus the dot product characterising each entry of $\boldsymbol{Q_1^T Q_2} = \boldsymbol{Q_1^T Z}$ is made by orthogonal vectors and is equal to zero.
